# *In vitro* reconstitution of the *Escherichia coli* 70S ribosome with a full set of recombinant ribosomal proteins

**DOI:** 10.1093/jb/mvab121

**Published:** 2021-11-08

**Authors:** Ryo Aoyama, Keiko Masuda, Masaru Shimojo, Takashi Kanamori, Takuya Ueda, Yoshihiro Shimizu

**Affiliations:** Laboratory for Cell-Free Protein Synthesis, RIKEN Center for Biosystems Dynamics Research, Suita, Osaka 565-0874, Japan; Department of Computational Biology and Medical Sciences, Graduate School of Frontier Sciences, The University of Tokyo, Kashiwa, Chiba 277-8562, Japan; Laboratory for Cell-Free Protein Synthesis, RIKEN Center for Biosystems Dynamics Research, Suita, Osaka 565-0874, Japan; Laboratory for Cell-Free Protein Synthesis, RIKEN Center for Biosystems Dynamics Research, Suita, Osaka 565-0874, Japan; Department of Computational Biology and Medical Sciences, Graduate School of Frontier Sciences, The University of Tokyo, Kashiwa, Chiba 277-8562, Japan; GeneFrontier Corporation, Kashiwa, Chiba 277-0005, Japan; Department of Computational Biology and Medical Sciences, Graduate School of Frontier Sciences, The University of Tokyo, Kashiwa, Chiba 277-8562, Japan; Department of Integrative Bioscience and Biomedical Engineering, Graduate School of Science and Engineering, Waseda University, Shinjuku, Tokyo 162-8480, Japan; Laboratory for Cell-Free Protein Synthesis, RIKEN Center for Biosystems Dynamics Research, Suita, Osaka 565-0874, Japan

**Keywords:** cell-free protein synthesis, protein translation, PURE system, ribosomal protein, ribosome assembly

## Abstract

Many studies of the reconstitution of the *Escherichia coli* small ribosomal subunit from its individual molecular parts have been reported, but contrastingly, similar studies of the large ribosomal subunit have not been well performed to date. Here, we describe protocols for preparing the 33 ribosomal proteins of the *E. coli* 50S subunit and demonstrate successful reconstitution of a functionally active 50S particle that can perform protein synthesis *in vitro*. We also successfully reconstituted both ribosomal subunits (30S and 50S) and 70S ribosomes using a full set of recombinant ribosomal proteins by integrating our developed method with the previously developed fully recombinant-based integrated synthesis, assembly and translation. The approach described here makes a major contribution to the field of ribosome engineering and could be fundamental to the future studies of ribosome assembly processes.

## Graphical Abstract

**Graphical Abstract:**
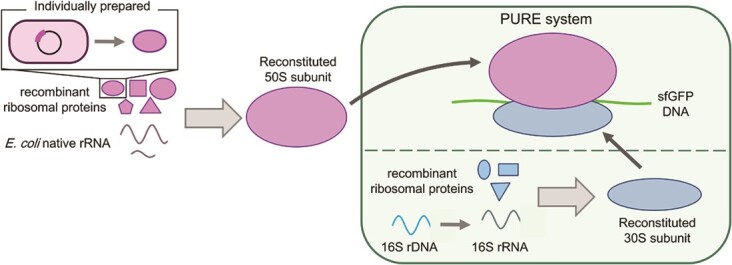

##  

As the major macromolecular complex in cells, the ribosome plays a central role in protein synthesis. In *Escherichia coli*, the 70S ribosome consists of two subunits: 50S and 30S. The 50S subunit comprises 2 ribosomal RNAs (5S and 23S rRNA) and 33 ribosomal proteins, which provides the peptidyl transferase centre that is essential for the peptide elongation process. The 30S subunit, which is composed of 1 ribosomal RNA (16S rRNA) and 21 ribosomal proteins, acts as the genetic decoding centre.

*In vitro* reconstitution studies of both *E. coli* ribosomal subunits have elucidated how these macromolecular complexes are formed by providing hierarchical assembly maps (*1**,**2*). Recently, studies using advanced technologies such as quantitative mass spectrometry (MS), cryo-electron microscopy and single molecule imaging have provided a more detailed view of the ribosome assembly processes (*3–7*).

Based on the knowledge of ribosome assembly pathways, ribosome engineering studies are also being developed by integration with a cell-free protein synthesis system. Development of the integrated synthesis, assembly and translation (iSAT) (*8*) has enabled integration of rRNA transcription, ribosome assembly and cell-free expression of test proteins, in a single reaction mixture. This technique has been utilized for evolutionary engineering of rRNA, particularly 16S rRNA, to design ribosomes with improved functions or altered chemical properties (*9**,**10*). A recent development using a set of recombinant ribosomal proteins for the 30S subunit fully recombinant-based iSAT (R-iSAT) has further advanced the engineering of ribosomal proteins (*11*). Because the ribosome is at the heart of self-replication in the central dogma process, these new technologies can provide a clue to answer fundamental biological questions such as ‘What is life?’ and ‘What is the origin of life?’ (*12–14*), in addition to the application of newly designed ribosomes to a field of protein engineering.

There are significant differences in our understanding of the assembly process *in vitro* between 30S and 50S subunit. In cells, ribosome assembly is mediated by a variety of ribosome biogenesis factors, including protein factors required for correct rRNA folding and enzymes required for post-transcriptional or post-translational modifications of rRNA and ribosomal proteins (*15*). These factors support the rapid and efficient supply of ribosomes in cells, which require <3 min during the exponential growth of *E. coli* cells (*16*). Nevertheless, a translationally active *E. coli* 30S subunit has been successfully assembled *in vitro* with a set of essential components without any ribosome biogenesis factors. *In vitro* transcribed 16S rRNA was shown to be assembled into the 30S subunit without any post-transcriptional modifications (*9**,**11**,**17*). A functional 30S subunit has also been assembled with a set of recombinant ribosomal proteins or *in vitro* synthesized ribosomal proteins without any post-translational modifications (*11**,**18–20*). Although biogenesis factors or some protein chaperones can facilitate the reconstitution efficiency (*11**,**19**,**21*), these observations suggest that the 30S subunit can be self-assembled autonomously from individual components.

Contrastingly, *in vitro* assembly of the *E. coli* 50S subunit is suggested to involve more complex processes than the 30S subunit. Although the iSAT experiments have demonstrated that a functionally active 50S subunit can be self-assembled with *in vitro* transcribed 5S and 23S rRNA (8), requirement of post-transcriptional modifications in 23S rRNA were suggested for the active 50S subunit assembly (*22*). Because the original iSAT experiments were performed in the presence of cell extracts, some unknown factors, presumably ribosome biogenesis factors, are thought to facilitate 50S subunit assembly. Several attempts have been made to use the *in vitro* transcribed rRNA for 50S subunit assembly. Reconstitution of active *Bacillus stearothermophilus* 50S subunit was successfully performed with *in vitro* transcribed 23S rRNA (*23*). Improvement of reconstitution efficiency of *E. coli* 50S subunit with transcribed 23S rRNA was observed by the addition of chemical substances such as osmolytes and antibiotics (*24*). These studies suggest that post-transcriptional modifications of a specific region in 23S rRNA are important for 50S assembly (*22*). Although it is unclear whether these modifications are required for the efficient formation or peptidyl-transferase activity of the 50S subunit, they are not universally conserved (*23**,**25*) and they may facilitate the conformational change of the 23S rRNA seen in cryo-electron microscopy analyses (*4*).

Although both cellular and cell-free expression methods have been used to prepare a set of ribosomal proteins for the *E. coli* 50S subunit (*20**,**26*), to our knowledge, there have been no reports of successful *in vitro* reconstitution of a functionally active 50S subunit. At present, *in vitro* reconstitution of this subunit requires the use of native components purified from mature 50S subunits, including native 5S and 23S rRNA and a mixture of ribosomal proteins, the so-called total proteins of the 50S (TP50) subunit. The protocol involves a two-step procedure: the first step involves 4 mM Mg^2+^ and incubation at 44°C, and the second step involves 20 mM Mg^2+^ and incubation at 50°C (*27*).

The use of individually prepared ribosomal proteins for ribosome assembly has the potential to reveal both the detailed mechanisms involved in the assembly processes and the individual functions of each ribosomal protein (*11*). Here, we established methods for preparing all 33 recombinant ribosomal proteins for the *E. coli* 50S subunit. The purified ribosomal proteins were mixed with native 5S and 23S rRNAs and processed using the two-step protocol described above. Sucrose density gradient centrifugation (SDGC) and liquid chromatography (LC)-MS analyses indicated successful formation of 50S particles containing all 33 of the prepared ribosomal proteins. The resultant reconstituted 50S subunit was translationally active in the PURE system, a reconstituted cell-free protein synthesis system (*28*), that utilizes the fluorescence of synthesized superfolder GFP (sfGFP) as a reporter. Finally, the reconstituted 50S subunit was applied to the previously developed R-iSAT (*11*) to form a 70S ribosome with a full set of 54 recombinant ribosomal proteins. In the R-iSAT reaction mixtures, the 30S subunit was assembled from *in vitro* transcribed 16S rRNA and 21 individually prepared recombinant 30S ribosomal proteins. The assembled 30S subunit subsequently bound to the reconstituted 50S subunit and sfGFP synthesis by the PURE cell-free expression system was observed. The approach described here will be useful in future ribosome engineering studies and analyses of ribosome assembly processes.

## Materials and Methods

### Preparation of native ribosomal components

*Escherichia coli* 70S ribosomes were prepared, as described previously (*29*). Separation of 70S ribosomes into 30S and 50S subunits was performed according to the previous report (*30*). SDGC was performed once for 5S and 23S rRNA preparation and twice for subunits preparation for protein synthesis experiments. A sucrose density gradient (12.2–36.6%) was formed in a centrifugation tube containing 10 mM Tris–HCl, pH 7.6, 10-mM magnesium chloride, 60-mM ammonium chloride, 400-mM sodium chloride and 7-mM 2-mercaptoethanol. The tubes were then centrifuged in a SW32 Ti rotor (Beckman Coulter) at 27,700 Rpm for 17 h (for 5S and 23S rRNA preparation) or in a SW28 Ti rotor (Beckman Coulter) at 27,000 Rpm for 14 h (for subunit preparation for protein synthesis experiments). The carry-over of other subunits was evaluated by *in vitro* sfGFP synthesis experiments where we verified that the protein synthesis activities with 50S subunits alone and 30S subunits alone were <1.5% and 0.02%, respectively, relative to those with equal amounts of 70S ribosomes. Preparation of TP50 was performed, as described previously (*31*). The 5S and 23S rRNAs were prepared from 50S subunits via phenol extraction. Purified 50S subunit fractions by SDGC were mixed with 0.12 volumes of 3 M potassium acetate, pH 5.2. The samples were mixed with 0.5 volumes of water-saturated phenol, vortexed for 8 min at 4°C, centrifuged at 10,000 *g* for 30 min, and then the aqueous phase was collected. This phenol extraction step was performed three times. After the third extraction step, the aqueous phase was mixed with 0.5 volumes of chloroform, centrifuged at 10,000 *g* for 30 min, aqueous phase was collected and rRNAs were precipitated by mixing the final aqueous phase with 0.5 volumes of 2-propanol and subsequent centrifugation at 10,000 *g* for 30 min. The pellets were rinsed with 70% ethanol and then dissolved in water. Resultant rRNA solution was treated with proteinase K by the addition of 1/20 volume of 20 mg/ml proteinase K Solution (Thermo Fisher Scientific) and subsequent incubation at 37°C for 30 min. The samples were again treated with water-saturated phenol and chloroform and then rRNAs were precipitated with 2-propanol. The pellets were rinsed with 70% ethanol, dissolved in water and the RNA concentration was determined by measuring the absorbance at 260 nm. The final 5S and 23S rRNA solutions were flash-frozen with liquid nitrogen and stored at −80°C until use.

### Preparation of recombinant proteins

Ribosomal proteins for the 30S subunit (bS1-bS21) and small ubiquitin-like modifier (SUMO)-specific Ulp1 protease were prepared, as described previously (*19*). Genes encoding the 33 large subunit ribosomal proteins, fused with a His-tagged SUMO protein at their N-terminus, were cloned into pET15b (Supplementary Data 1; Supplementary Table 1) and transformed into an *E. coli* Rosetta(DE3)pLysS strain. Cells were grown in LB medium containing 100 μg/ml ampicillin and 25 μg/ml chloramphenicol to an OD_660_ of 0.3–1.0 at 37°C, followed by induction of overexpression with 0.1 mM isopropyl-β-D-thiogalactoside (the concentration was changed to 0.25 mM for bL33, and to 0.5 mM for uL3, uL5, uL10, uL16 and bL35), 3 h of cultivation at 37°C and recovery of cells with centrifugation. The protocol used to purify each ribosomal protein, including the purification steps and buffers applied, depended on the specific characteristics of the protein (Supplementary Data 2). The harvested cells were disrupted by sonication at 4°C in lysis buffer. Resultant lysates were centrifuged at 20,000 *g* for 45 min. Supernatants were basically recovered except for uL5 and bL20, which were insoluble in the applied lysis buffer. For uL5 and bL20, the pellets were solubilized with buffer C5 containing 8-M urea and the supernatants were recovered after the centrifugation at 7000 *g* for 30 min. Ni^2+^-chelate affinity purification was performed as follows. For batch purification of proteins using Ni-NTA agarose (QIAGEN) or complete His-tag Purification Resin (Roche), His-tagged proteins were bound to the resin, washed with 50 column volumes of 98% basal buffer and 2% elution buffer and eluted with 40% basal buffer and 60% elution buffer. For high performance liquid chromatography purification of proteins processed using HisTrap HP (Cytiva), His-tagged proteins were bound to the resin, washed with 10 column volumes of 95% basal buffer and 5% elution buffer and eluted with a linear gradient from 95% basal buffer and 5% elution buffer to 40% basal buffer and 60% elution buffer. To increase the purities of the purified proteins, multiple rounds of Ni^2+^-chelate affinity purification were performed for some proteins including uL5, uL10, uL15, bL17, uL29, bL31 and bL36. Fractions containing the target protein were recovered and dialyzed against basal buffer in the subsequent purification process and then further purified as described. Fractions containing the target protein were recovered for protease digestion. When the urea concentration of the resultant solution was 6 M (uL5, uL10, uL15 and bL20), it was dialyzed against buffer C3 for 3 h and then dialyzed against buffer C1 for 30 min, followed by the addition of active domain of yeast Ulp1p (Δ1–402) (*32*). The dialysis against buffer C1 was continued at 4°C overnight to cleave the SUMO moiety. For other proteins, active domain of Ulp1p was directly added to the recovered fractions and then the resultant mixtures were dialyzed against digestion buffer at 4°C overnight. After the digestion, solution of some proteins was dialyzed against recovery buffer to secure the proteins from forming aggregation. Cleaved His-tagged SUMO moiety was removed by Ni^2+^-chelate affinity purification. The protein solutions were flowed into Ni^2+^ resin equilibrated with basal buffer and flow through was recovered and dialyzed against dialysis buffer. Resultant solutions were further purified with anion- and/or cation-exchange chromatography. Obtained proteins were concentrated by Amicon Ultra 10 k or 3 k (Merck Millipore) or by using ammonium sulphate precipitation. When the ammonium sulphate precipitation is performed, the purified protein solution containing 80% saturated concentration of ammonium sulphate were centrifuged at 32,000 Rpm (119,000 *g*) for 30 min at 4°C in a Type 45Ti rotor (Beckman Coulter). The precipitated proteins were resuspended by resuspension buffer. Obtained purified protein solutions were sequentially dialyzed against dialysis buffers 1–6. After precipitants were removed by centrifugation at 20,000 *g* for 10 min, protein concentrations were determined by Bradford or BCA method and the proteins were stored at −80°C. A gene encoding LgBiT, large N-terminal region of the split NanoLuc system (*33*), was cloned into pET15b (Supplementary Data 1) by transferring the gene from LgBiT Expression Vector (Promega). Purification of LgBiT by Ni^2+^-chelate affinity purification was performed similarly with the previous protocol (*34*).

### Native MS analysis

Native MS analysis was performed using an Orbitrap mass spectrometer (Q Exactive; Thermo Fisher Scientific) equipped with a nanospray ion source (Nanospray Flex, Thermo Fisher Scientific). Purified ribosomal proteins uL5-bL36 were desalted by using self-prepared stage tips (*35*) and eluted with 50% acetonitrile containing 0.1% acetic acid. uL1-uL4 were dialyzed against water overnight and they were mixed with an equal volume of a solution containing 50% acetonitrile and 1% acetic acid. The samples were filled into the Cellomics tips (CT-10 μm, HUMANIX) and electrosprayed at spray voltage 1.7 kV into the MS (positive mode, scan range of 400–2000 *m/z*, 140,000 FWHM resolution). Theoretical spectra were obtained using Xcalibur (Thermo Fisher Scientific). Deconvolution analysis was performed with TopPIC Suite (*36*) using default parameters.

### 50S subunit assembly

Equal amounts of each of the 32 ribosomal proteins except for bL12 were mixed with four times the amount of bL12 and dialyzed against buffer V (20-mM Tris–HCl, pH 7.6, 4-mM magnesium chloride, 400-mM ammonium chloride, 6-M urea, 7-mM 2-merchaptoethanol) overnight at 4°C. They were further dialyzed against buffer IV (20-mM Tris–HCl, pH 7.6, 4-mM magnesium chloride, 400-mM ammonium chloride, 7-mM 2-merchaptoethanol) for 45 min at 4°C for three times. The concentration of the obtained protein mixture was determined according to the absorbance at 230 nm, where 1 A_230_ unit was regarded to be equivalent to TP50 obtained from 360 pmol of ribosomes (*31*). The assembly procedure was as described (*31*). Briefly, the assembly mixtures (250 μl) contained 20-mM Tris–HCl, pH 7.4, 4-mM magnesium acetate, 400-mM ammonium chloride, 0.2-mM EDTA, 5-mM 2-mercaptoethanol, 6.25 A_260_ units of 5S and 23S rRNA (240 pmol) and 0.75 A_230_ units (270 pmol) of 33 recombinant proteins mixture or TP50. They were incubated at 44°C for 20 min and then, the magnesium acetate concentration was increased to 20 mM and the mixtures were further incubated at 50°C for 90 min. The reconstituted products were concentrated and buffer exchanged to ribosome buffer (10-mM Hepes-KOH, pH 7.6, 10-mM magnesium acetate, 30-mM potassium acetate and 1-mM dithiothreitol) with Amicon Ultra 0.5-ml centrifugal filter units, MWCO 30KDa (Merck Millipore). The concentration of the obtained 50S subunits was determined according to the absorbance at 260 nm (1 A_260_ unit was regarded to 36 pmol (*31*)).

### Analysis of 50S subunit assembly

50S subunit assembly reaction mixtures (100 μl) were directly layered onto a centrifugation tube containing solution (20-mM Tris–HCl, pH 7.6, 4-mM magnesium chloride, 400-mM ammonium chloride and 7-mM 2-mercaptoethanol) with sucrose density gradient (5–20%). The tubes were centrifuged with SW32 Ti rotor (Beckman Coulter) at 30,600 Rpm for 5 h. The solution was fractionated from the top by automatic liquid charger CHD255AA (Advantec) with continuously monitoring the absorbance at 254 nm. For LC–MS analysis, fractions containing 50S subunits were pooled, mixed with 1/10 volume of 50% trichloroacetic acid and centrifuged at 10,000 *g* for 30 min and the pellets were rinsed with cold acetone. Enzymatic digestion was performed basically according to a phase transfer surfactant (PTS)-aided protocol (*37*). The pellets were dissolved in 10 μl of a PTS buffer (10-mM sodium deoxycholate, 10-mM sodium N-lauroylsarcosinate and 50-mM NH_4_HCO_3_), reduced with 10-mM TCEP at 37°C for 30 min, alkylated with 20-mM iodoacetamide at 37°C for 30 min and quenched with 20 mM L-cysteine residues. Before the digestion, samples were diluted 5-fold with 50 mM NH_4_HCO3. Digestion was performed by adding 100 ng of Lys-C (FUJIFILM Wako Chemicals) at 37°C overnight. After the digestion, 5 μl of 10% TFA was added to the sample to precipitate the detergents. After the sample was centrifuged at 15,000 *g* for 5 min at 4°C, supernatant was desalted by using self-prepared stage tips (*35*) and dried with SpeedVac. Mass spectrometric analysis was performed using an Orbitrap mass spectrometer (LTQ OrbitrapVelos Pro, Thermo Fisher Scientific) equipped with a nanospray ion source (Nanospray Flex, Thermo Fisher Scientific) and a nano-LC system (UltiMate 3000, Thermo Fisher Scientific). The dried peptide mixtures were dissolved in a solution containing 5% acetonitrile and 0.1% TFA, and ~1 pmol of each sample was applied to the nano-LC system. Peptides were concentrated using a trap column (0.075 × 20 mm, 3 μm, Acclaim PepMap 100 C18, Thermo Fisher Scientific) and then separated using a nanocapillary column (0.1 × 150 mm, 3 μm, C18, Nikkyo Technos) using two mobile phases A (0.1% formic acid) and B (acetonitrile and 0.1% formic acid) with a gradient (5% B for 5 min, 5–45% B in 40 min, 45–90% B in 1 min and 90% B in 4 min) at a flow rate of 500 nL/min. Elution was directly electrosprayed (2.2 kV) into the MS (positive mode, scan range of 200–1500 *m/z*, 60,000 FWHM resolution). Data analysis was performed using Proteome Discoverer 2.2 (Thermo Fisher Scientific) to select peptides for the semi-quantitative analysis. Peptides with sufficient peptide spectrum matches (PSMs) were selected and quantified using Skyline (*38*). Peak area was calculated with setting MS1 filter to a count of three (M, M + 1 and M + 2).

### Protein synthesis

DNA sequence for the synthesis of small C-terminal peptide of the split NanoLuc system (HiBiT-tag) (*33*) was cloned into pET15b (Supplementary Data 1), in which the ORF contained MIIIIGSSG peptide sequence fused with the HiBiT-tag sequence (VSGWRLFKKIS). DNA templates for 16S rRNA transcription and sfGFP synthesis were obtained from our previous study (*11*). Cell-free protein synthesis reaction mixtures (5 μl for HiBiT-tag synthesis and 10 μl for sfGFP synthesis) contained solutions I (Buffer Mix) and II (Enzyme Mix) of PUREfrex 2.0 (GeneFrontier Corporation), 1-nM HiBiT-tag or sfGFP DNA template, 50 nM (HiBiT-tag synthesis) or 200 nM (sfGFP synthesis) *E. coli* native 30S subunit and 50 nM (HiBiT-tag synthesis) or 200 nM (sfGFP synthesis) *E. coli* native 50S subunit or reconstituted 50S subunit. Externally added Mg^2+^ concentration except for PUREfrex solutions was adjusted to be 4 mM by adding magnesium acetate solution. HiBiT-tag synthesis was detected with Nano-GloR Luciferase Assay System (Promega). After HiBiT-tag synthesis at 37°C for 12 h, 0.6-μl aliquots were mixed with 50 pmol purified LgBiT protein contained in 25 μl incubation buffer (20-mM Hepes-KOH, pH7.6, 100-mM potassium chloride, 30% glycerol and 5-mM dithiothreitol). After incubation at 37°C for 5 min, they were further mixed with 25-μl Nano-GloR Luciferase Assay Reagent (Promega). In total, 40 μl of the sample was added onto a white 96-well plate and the luminescence was immediately measured with GloMax Explorer Multimode Microplate Reader (Promega). Time-lapse sfGFP synthesis was detected by measuring the fluorescence by Stratagene Mx3005P (Agilent Technologies) at 37°C for 12 h. Integration with previously developed R-iSAT was performed according to the previous report (*11*). Reaction mixtures (10 μl) contained solutions I (buffer mix) and II (enzyme mix) of PUREfrex 2.0 (GeneFrontier Corporation), 5-nM sfGFP DNA template, 20-nM DNA template for 16S rRNA transcription, 500-nM each ribosomal proteins bS1-bS21 and 200-nM reconstituted 50S subunit. Externally added Mg^2+^ concentration except for PUREfrex solutions was adjusted to be 4 mM by adding magnesium acetate solution. Time-lapse sfGFP synthesis was detected by measuring the fluorescence by Stratagene Mx3005P (Agilent Technologies) at 37°C for 12 h.

## Results

### Purification of 33 ribosomal proteins for the 50S subunit

To purify the 33 ribosomal protein components of the *E. coli* 50S subunit (Supplementary Table 1), we followed our previous protocol used for reconstitution of the 30S subunit (*19*). Briefly, each 50S ribosomal protein was fused with an N-terminal His-tagged SUMO protein at its N-terminus (Supplementary Data 1) and then recombinantly expressed in *E. coli* cells. All proteins were basically processed in a similar manner, in which Ni^2+^-chelate affinity purification, protease cleavage of SUMO moiety (*32*), removal of SUMO moiety with Ni^2+^ column chromatography and ion exchange chromatography were sequentially performed. However, as reported previously (*26*), many of the ribosomal proteins were insoluble in regular buffer conditions, and thus, they were occasionally processed under denaturing conditions using urea according to their properties (Supplementary Data 2). To our surprise, with the exception of bL20, all other purified proteins were finally solubilized in a high-salt non-denaturing buffer containing 1 M KCl. The bL20 protein was stored in a buffer containing 3 M guanidine hydrochloride. As high concentration of ions and guanidine hydrochloride can inhibit reconstitution experiments, they were removed by dialysis prior to subsequent experiments (see Experimental Procedures).

**Fig. 1:**
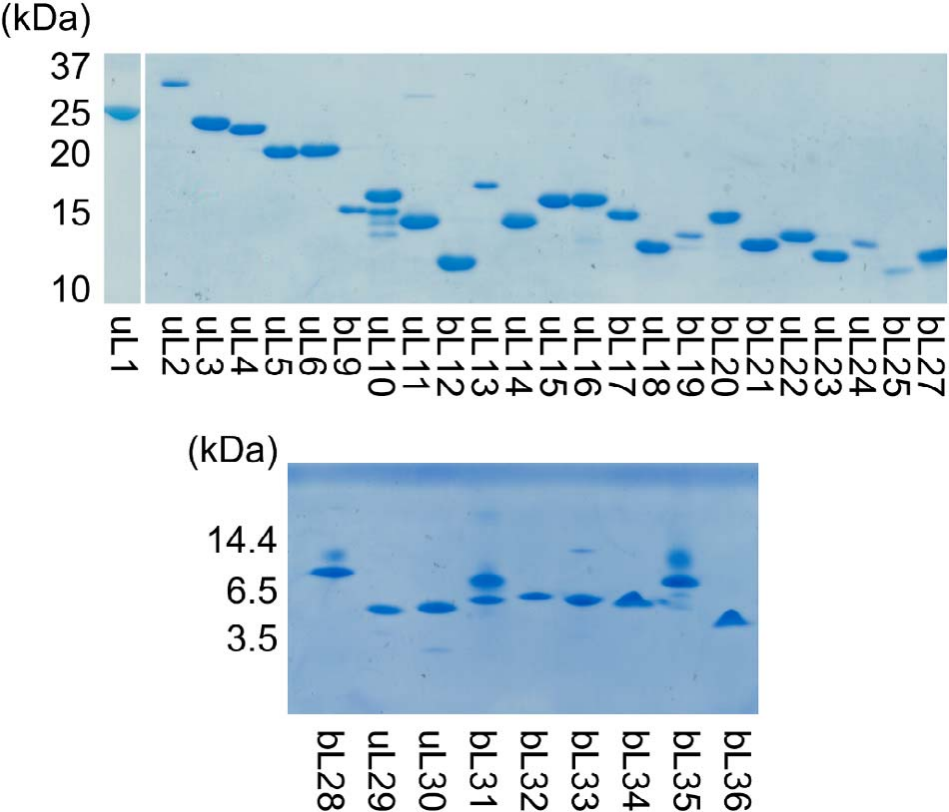
**Purified recombinant ribosomal proteins for the 50S subunit.** The uL1, uL2-bL27 and bL28-bL36 were analysed with 15% and 17% SDS-PAGE and 16.5% Urea-Tris/Tricine-SDS-PAGE, respectively. The gels were stained with Coomassie Brilliant Blue G-250.

The purified ribosomal proteins displayed high homogeneity in an sodium dodecyl sulfate polyacrylamide gel electrophoresis (SDS-PAGE) analysis (Fig. 1). However, minor bands were observed for the uL10 and bL31 fractions. It was reported previously that overexpression of uL10 in *E. coli* cells causes its rapid degradation by several regulatory proteins (*39*), which was also seen in our experiment. Co-expression with bL12 might improve the purity of uL10 in future experiments. The bL31 protein seemed to be degraded through repeated freeze thaw processes; therefore, we avoided the use of repeatedly frozen and thawed bL31 in the reconstitution experiments.

### Native MS analysis of the 33 purified 50S ribosomal proteins

The 33 ribosomal proteins were further analysed using native MS. With the exceptions of uL2 and bL31, the MS signals of all other proteins matched the theoretical mass distributions of proteins without post-translational modifications (Supplementary Data 3). Deconvoluted values calculated with available software (*36*) showed almost same values with each monoisotopic molecular weight of the ribosomal proteins (maximum shift of 2 Da), suggesting acquisition of proteins in the expected forms (Supplementary Table 1; Supplementary Data 4). Signals for uL2 could not be detected possibly due to its low ionization efficiency. Signals for bL31 were slightly shifted from the theoretical counterparts (Supplementary Data 3) and the deconvoluted value showed shift of 1 Da (Supplementary Table 1; Supplementary Data 4). Except for bL31, the deconvoluted values of other proteins <10,000 Da (bL27-bL36) matched the monoisotopic molecular weight of each protein without any shifts, suggesting accuracy of the deconvolution within this range. These observations suggest the partial formation of disulphide bonds (Supplementary Fig. 1a). It has been suggested previously that a maximum of two disulphide bonds are formed in bL31 by four cysteine residues at positions 16, 18, 37 and 40, which are located close to each other in the 3D structure (Supplementary Fig. 1b) (*40**,**41*).

Five 50S ribosomal proteins are reportedly post-translationally modified (uL3: methylation; uL11: tri-methylation of three residues; bL12: methylation and acetylation; uL16: methylation; bL33: methylation) (*42*). A native MS analysis of these purified proteins (Supplementary Data 5) showed that uL3 and uL11 were partially methylated but bL12 and bL33 were not methylated. The mass distribution of the purified uL16 protein was similar to that of its methylated counterpart (+14). However, the distribution of oxidized uL16 (+16 and +32) were closer distribution to the experimental results, suggesting that the mass shifted peaks of the purified uL16 protein might reflect single or double oxidization. The analysis revealed that purified bL12 was either not acetylated or acetylated in very small amounts. One of the methylation sites of uL11 (Ala1), the acetylation site of bL12 and the methylation site of uL16 and bL33 are at the N-terminal amino group and our purification method of the ribosomal proteins blocks this group by SUMO fusions. Therefore, it is probable that these sites are not modified during the overexpression of the proteins in cells.

The degrees of methylation of the purified uL3 and uL11 proteins were examined further by comparing their native MS profiles with those of their post-translationally methylated counterparts, which were prepared by co-expression with specific methyltransferases PrmB (uL3) and PrmA (uL11) (*42*). This analysis (Supplementary Data 5) showed that uL3 co-expressed with PrmB was fully methylated. The degree of methylation of uL11 was increased when it was co-expressed with PrmA, although it was still not fully methylated, possibly due to the absence of methylation at its N-terminus (Ala1). These findings indicate that the purified uL3 and uL11 proteins were not fully methylated and are consistent with our previous study, in which large parts of purified recombinant ribosomal proteins were unmodified (*11*).

### Reconstitution of the 50S subunit with 33 purified ribosomal proteins

**Fig. 2:**
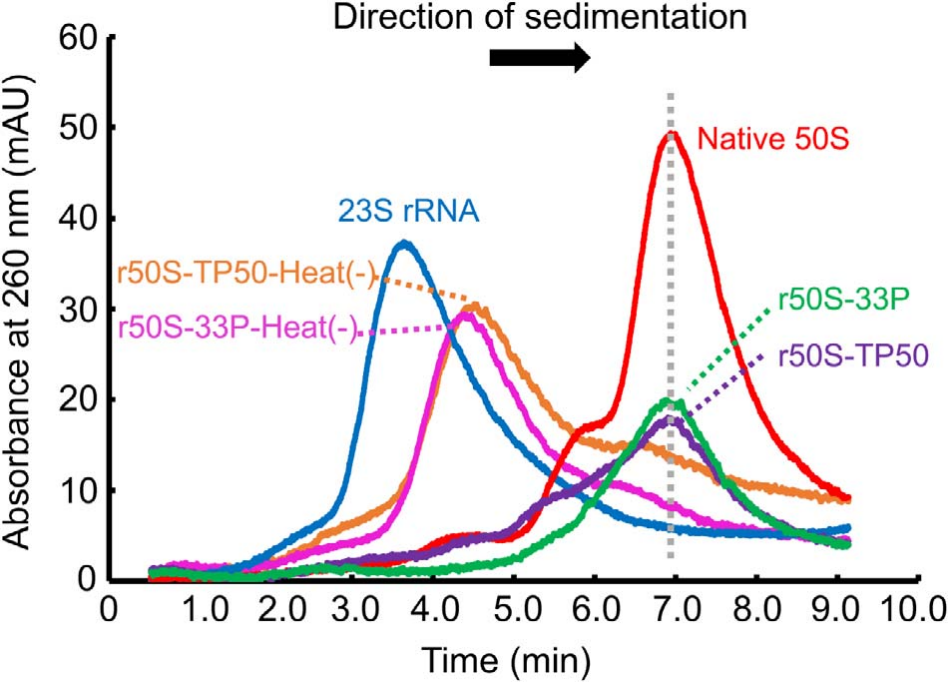
**SDGC analysis of reconstituted particles.** Native 50S subunits, reconstituted subunits with TP50 (r50S-TP50 and r50S-TP50-Heat(−)) and 33 recombinant ribosomal proteins (r50S-33P and r50S-33P-Heat(−)) and native 5S and 23S rRNA mixture were analysed. The r50S-TP50 and r50S-33P were prepared by the normal procedure and r50S-TP50-Heat(−) and r50S-33P-Heat(−) were prepared without heat activation, in which all steps were performed on ice.

Reconstitution of the 50S subunit was addressed by SDGC analysis. The 33 purified ribosomal proteins (33P) were assembled with rRNAs (5S and 23S rRNA) obtained from native 50S subunits. As a control experiment, TP50 obtained from native 50S subunits was also evaluated. We adopted a two-step protocol developed by the Nierhaus group (*27**,**31*), where the assembly proceeds under low magnesium (4 mM) conditions at 44°C in the first step, and then under high magnesium (20 mM) conditions at 50°C in the second step. The reconstituted particles with both 33P and TP50 showed similar sedimentation with the native 50S subunits (Fig. 2). The top peaks of both particles were almost identical to that of the native 50S subunits, suggesting the assembly successfully proceeded to the 50S particle formation, not the intermediates such as 33S, 41S and 48S particles, known to be formed during the assembly (*4*). By contrast, when the reconstitution was performed on ice, smaller particles, possibly representing 33S particles, were accumulated, which is consistent with a previous observation (*43*). Almost equimolar amounts of rRNAs (240 pmol) and ribosomal proteins (270 pmol each) were used in this experiment. Judging from the fact that only a single peak was seen in the sedimentation profiles of both reconstituted particles, most of the added rRNAs and proteins were estimated to interact with each other; however, some proteins were found to be less assembled according to the LC–MS analysis (see below).

**Fig. 3:**
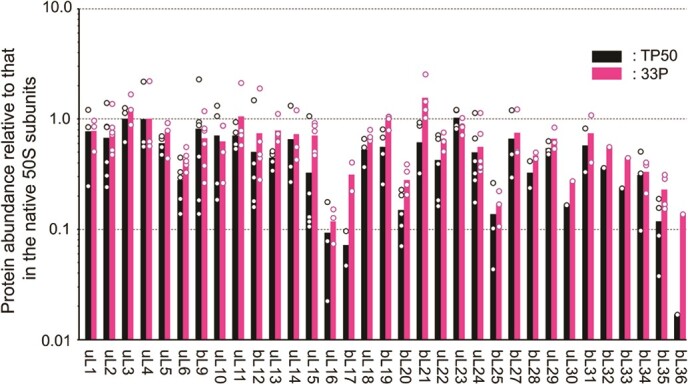
**LC**–**MS analysis of reconstituted 50S subunits.** The ribosomal proteins in fractions separated via SDGC were analysed with LC–MS in a semi-quantitative manner. Recombinant 50S subunits were generated using 33P or TP50. The ratio of the signal intensity of each peptide peak relative to that of the corresponding peptide in the native 50S subunit was calculated. The ratios were then normalized to the average uL4 peptide signal. Each dot represents the signal intensity ratio of an individual peptide listed in Supplementary Data 7.

Next, the proteins in the SDGC fractions of the native 50S subunits and the 50S particles formed with 33P and TP50 were analysed via LC–MS (Fig. 3). The analysis was performed in a semi-quantitative manner, in which the signal intensity of each peptide peak in the recombinant 50S particles was normalized to that of the corresponding peptide in the native 50S subunits. The ratios were then normalized further to the average of uL4 peptide peaks, which is one of the primary binding proteins directly associates with 23S rRNA. Because uL4 plays a crucial role in early assembly (*44*), we assumed that the stoichiometry of uL4 binding to 23S rRNA remains unchanged during the early to late assembly process. The selection of the peptides for the analysis were performed according to the number of PSM, which represents the total number of peptide identification by tandem MS and correlates with the signal intensities of the detected peptide, for reliable semi-quantification (Supplementary Data 6). The peptides containing methionine residues were omitted from the analysis because they have negative effect on the semi-quantification by their oxidation.

After normalization, we noticed a prominent difference between the normalized signals of the bL31 peptide in 50S with TP50 and 50S with 33P. Three peptide peaks (CHPFFTGK, QRDVATGGRVDRFNK and RFNIPGSK) were quantified for bL31. For the two former peptides, the normalized ratios were similar between two particles (Supplementary Data 7). However, the intensity of RFNIPGSK peptide in particles reconstituted with 33P was more then 50-fold higher than that in particles reconstituted with TP50. It is known that bL31 undergoes C-terminal processing, mediated by protease 7 (OmpT), which removes RFNIPGSK from its C-terminus, during sample preparation (*42**,**45*). This finding may indicate that the major forms of bL31 in the native 50S subunits and TP50 preparations were C-terminus deleted forms and that the addition of recombinant 33P generated the 50S subunit with the non-cleaved recombinant bL31 protein. This prominent difference between the bL31 peptides in the three forms of 50S examined here suggests that the recombinant ribosomal proteins participated in the assembly reactions.

The LC–MS result (Fig. 3) showed that the levels of proteins in the TP50- and 33P-generated 50S subunits were more or less comparable with those in the native 50S subunits. However, the levels of some proteins, such as uL16, bL17 and bL36, were lower in the reconstituted subunits than in the native form, particularly for the TP50-generated subunits. A recent study that limited the cellular bL17 expression showed interesting features, including the accumulation of incomplete 50S particles and upregulation of ribosome assembly factors, suggesting this protein plays a key role in the 50S subunit assembly process (*46*). The uL16 and bL36 proteins are categorized as the late assembly proteins of the 50S subunit. The uL16 protein reportedly triggers conformational changes of 23S rRNA helix H89, an integral part of the peptidyl transferase centre (*4*). The bL36 was reported to recruit and stabilize uL16 by fixing H89 in the proper position (*47*). Thus, increasing the amounts of these proteins in the assembled subunits might be important factors for establishing efficient subunit assembly system in the future. Nevertheless, the data presented here suggest that all 33 of the recombinant ribosomal proteins were successfully incorporated into the reconstituted particles, in a similar way as when using TP50.

### Protein synthesis activity of the reconstituted 50S subunits

**Fig. 4:**
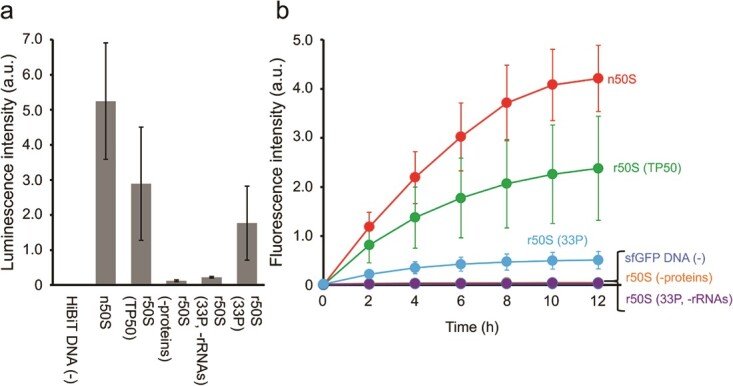
**Protein synthesis activities of reconstituted 50S subunits.** Protein synthesis activities of native 50S subunits (n50S), reconstituted subunits with TP50 (r50S (TP50)) and 33 recombinant ribosomal proteins (r50S (33P)), reconstituted subunits only with 5S and 23S rRNA mixture (r50S (−proteins)) and only with 33 proteins (r50S (33P, -rRNAs)) were measured by performing HiBiT-tag peptide synthesis (a) and sfGFP synthesis (b) in the PURE system. End-point luminescence intensity exhibited by the interaction between LgBiT protein and synthesized HiBit-tag (a) and time-lapse changes of fluorescence intensity of synthesized sfGFP (b) are shown.

Activities of the reconstituted subunits were measured in the PURE system (*28*). Both short peptide synthesis and regular size protein synthesis were examined using a split NanoLuc system (*33*) and fluorescence measurement of sfGFP, respectively. They were addressed by replacement of ribosomes with native 30S subunits and reconstituted 50S subunits in the PURE system. The split NanoLuc system comprises two fragments: a large N-terminal region (LgBiT) and a small C-terminal peptide. The HiBiT-tag (VSGWRLFKKIS) is one of the developed small C-terminal peptides that exhibits increased affinity for LgBiT (*33*) and was applied in this study. The tag was synthesized with the reconstituted 50S subunits and the evaluation was performed by luminescence measurement emitted by the interaction with LgBiT. The result showed similar level of luminescence with the reconstituted 50S subunits using 33P, compared with those using TP50, demonstrating functional reconstitution of the 50S subunits (Fig. 4a). Time-lapse measurements of sfGFP synthesis also showed formation of translationally active 50S subunits with 33P, although the activity was lower than those of the native 50S subunits and those generated using TP50 (Fig. 4b).

There remains difference between TP50- and 33P-dependent 50S subunits in both peptide and protein synthesis activity. One of the major differences between TP50 and 33P is that many of the recombinant ribosomal proteins lack post-translational modifications (Supplementary Data 5). Enzymes responsible for the methylation of uL3 (PrmB) and uL11 (PrmA) and the acetylation of bL12 (RimL) have been identified (*42*). The amino acid residues of uL11 that are methylated by PrmA are located in the N-terminal domain, in proximity to the binding sites of elongation factors G and Tu. PrmB reportedly affects the ribosome assembly process. By contrast, acetylated bL12 is accumulated in the stationary phase of *E. coli* growth (*48*) and a mutant strain in which bL12 remains deacetylated is not phenotypically different to the wild-type strain (*49*). In view of these findings, we formed recombinant 50S subunits using methylated forms of uL3 and uL11 (Supplementary Data 5). However, time-lapse measurements of sfGFP synthesis revealed no differences between the activities of the subunits containing methylated and unmodified uL3 and uL11 proteins (Supplementary Fig. 2), suggesting that other factors are responsible for the observed differences between the activities of the TP50- and 33P-generated 50S subunits. This aspect should be elucidated in the future.

### Total reconstitution of the 70S ribosome from a complete set of recombinant ribosomal proteins

**Fig. 5:**
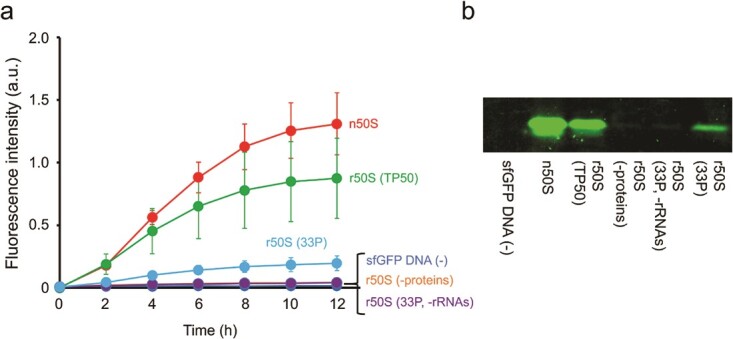
**Integration with the R-iSAT.** Protein synthesis activities of reconstituted ribosomes using native 50S subunits (n50S), reconstituted subunits with TP50 (r50S (TP50)) and 33 recombinant ribosomal proteins (r50S (33P)), reconstituted subunits only with 5S and 23S rRNA mixture (r50S (−proteins)) and only with 33 proteins (r50S (33P, -rRNAs)) were measured by performing sfGFP synthesis in the R-iSAT. Time-lapse changes of fluorescence intensity of synthesized sfGFP (a) and fluorescent images of sfGFP in 15% SDS-PAGE (b) are shown.

Finally, we integrated reconstitution of the 50S subunits with the previously developed R-iSAT for small ribosomal subunits assembly to form a reconstituted 70S ribosome (*11*). A total of 54 recombinant ribosomal proteins were used in this scheme (Supplementary Fig. 3), in which the 30S subunits were formed with co-transcriptionally synthesized 16S rRNA and 21 ribosomal proteins and then bound with the reconstituted 50S subunits formed by the two-step protocol described above. As a result, an sfGFP signal was detected for reconstituted ribosomes formed using 50S subunits generated with 33P (Fig. 5a). An SDS-PAGE analysis of synthesized product showed the synthesis of full-length sfGFP (Fig. 5b), demonstrating successful reconstitution of 70S ribosomes from a complete set of recombinant ribosomal proteins for both subunits.

## Discussion

The present study demonstrates total reconstitution of functionally active *E. coli* 50S subunits, as well as 70S ribosomes, from sets of recombinant ribosomal proteins. In addition to the previously described generation of 21 proteins for the reconstitution of 30S subunits (*11**,**19*), we have described the similar preparation of 33 recombinant proteins for the reconstitution of 50S subunits using the SUMO protein fusion technique (*32*). Introduction of a His-tag at the N-terminus of the SUMO protein enabled affinity purification of all 33 ribosomal proteins for increasing homogeneity (Fig. 1). In addition, as discussed previously (*19*), the SUMO protein fusion technique permits the preparation of proteins with any N-terminal amino acid residue except proline. The SUMO protein-specific protease cleaves immediately after the SUMO protein, without leaving any fragments; however, this process is inhibited when a proline residue is located just after the cleavage site. In our present study, the proline residue at the N-terminus of bL35 was replaced with alanine to ensure efficient cleavage, as with the previous study (*19*). The N-termini of other proteins are successfully designed to be consistent with previous observation using MS analysis (*50*), which were confirmed with our native MS analysis (Supplementary Data 3 and 4). The strategy adopted here may be useful for reconstitution of other multi-molecular complexes, in which the presence of affinity tags inhibits structure formation.

Whereas cellular or cell-free expression and preparation of a set of ribosomal proteins for the 50S subunit has already been reported (*20**,**26*), to our knowledge, reconstitution of the 50S subunit from recombinant ribosomal proteins has not been achieved to date. By contrast, *in vitro* reconstitution of the 50S subunit from native components, including TP50 and 23S and 5S rRNAs, was achieved about half a century ago (*27*). Additionally, co-transcriptional reconstitution of the 50S subunit from TP50 and DNAs encoding 23S and 5S rRNAs was developed as the iSAT (*8*). Compared with these studies, our present study provides a new research direction using recombinant ribosomal proteins as a substitute for TP50. This process may enable studies of the specific functions of individual ribosomal proteins in both the assembly and translation processes.

The SDGC analysis showed that the major form of particles reconstituted using the 33 recombinant proteins was 50S, as observed for particles reconstituted using TP50 (Fig. 2). An LC–MS analysis also showed similar abundances of recombinant proteins in the 33P- and TP50-generated 50S subunits (Fig. 3). However, the abundances of some proteins, including uL17 and bL36, were greater in the 33P-generated subunits than in the TP50-generated subunits, suggesting that recombinant proteins are more effective for the subunit assembly than TP50. Notably, we found that, unlike bL31 in the TP50-generated subunit, recombinant bL31 in the 33P-generated subunit was intact without C-terminal deletion. A recent study showed that the intact bL31 can counteract intrinsic destabilization by bridging the two ribosomal subunits (*51*), and thus, a recombinant protein strategy may have a positive effect on the functionality of the reconstituted 50S subunits.

Nevertheless, the reconstituted 50S subunits generated using recombinant proteins were less active than those generated using TP50. This difference was more pronounced for sfGFP synthesis than for short peptide synthesis (Fig. 4). One of the major differences between recombinant proteins and TP50 is the lack of post-translational modifications (Supplementary Data 5), and therefore, we methylated uL3 and uL11 in 33P using previously identified modification enzymes, in which we could not observe any improvements (Supplementary Fig. 2). The enzymes responsible for post-translational modifications of uL16 and bL33 have not yet been identified. It should be noted that the methylation of uL16 and bL33 are introduced at the N-terminal amino group. Three of nine methylation of uL11 are also introduced at the N-terminus. Our preparation method is based on the N-terminal fusion of the SUMO protein, and therefore, methylation of these proteins should be carefully designed, *e.g.* the development of the *in vitro* methylation method of purified proteins. Thus, future effort of introducing these modifications may increase the activity of reconstituted 50S subunits.

The differences between the activities of 33P- and TP50-generated 50S subunits could also be attributed to carry-overs of some biogenesis factors in TP50. Although the ribosome was purified to homogeneity by integration of hydrophobic chromatography and sucrose cushion-based ultracentrifugation (*29*), it is still possible that some biogenesis factors are tightly bound to the ribosome and carried over in final TP50 preparations. Such factors might increase the activity of the TP50-dependent subunits, whereas such support is missing in recombinant protein-dependent reconstitution. Another reasons for the discrepancy could be subtle differences between the conformations of native and recombinant proteins, although further experiments are required to investigate this possibility.

Bottom-up construction of a self-replicating synthetic cell is one of the important and challenging milestones in the field of synthetic biology (*12–14*). Recent advancements have allowed partial realization of this goal by addressing self-regeneration of the PURE system components in solution- or microfluidics-based cell-free reaction systems (*52**,**53*). The present achievement of the ribosome reconstitution from the full set of recombinant proteins, together with our recent report showing protein expression using *in vitro* transcribed tRNAs (*54*), may expand the target of self-regeneration to the ribosomes and tRNAs, making fundamental steps to the complete bottom-up construction of self-replicable artificial cells. However, unlike the 30S subunit, which has been successfully reconstituted with a set of recombinant proteins and *in vitro* transcribed 16S rRNA (*11*), *E. coli* 50S subunit reconstitution with *in vitro* transcribed 23S rRNA has not been achieved to date except for iSAT utilizing cell-extracts for the reconstitution reaction (*8*). Importance of post-transcriptional modifications in the specific region of 23S rRNA has been suggested (*22*) and clarifying this point based on the present study may be crucial for the ultimate goal of the bottom-up construction of a self-replicating synthetic cell.

In this study, protein synthesis activity is achieved using a total of 90 recombinant proteins, including 36 PURE system components, 21 30S ribosomal proteins and 33 50S ribosomal proteins (Fig. 5). Controlling the compositions and stoichiometries of these proteins is essential for elucidating the function of individual proteins and/or engineering of the reaction system (*11*) and for addressing self-regeneration of the system components (*53*). To this end, application of the recently developed microbial ‘consortia’ (*55*) or OnePot strategy (*56*) would be beneficial for preparing and dealing with such a large number of proteins. Although the protocol used to prepare the 50S subunit ribosomal proteins described here depended on each protein’s characteristics (Supplementary Data 2), it would be probable to prepare them in a combined manner similarly with TP50 preparation by solubilizing proteins by urea and removing it before the reconstitution experiments, which should be addressed in the future. Overall, the results and procedures described here may contribute to the goal of constructing a self-replicable synthetic cell and may shed light on the origin, function and assembly principle of the ribosome, one of the most fundamental multi-molecular complexes that is closely related to the origin of life.

## Supplementary Material

Web_Material_mvab121

## Data Availability

All the data for this manuscript is supplied as Supplementary Data.
