# Supplementary material for: In vitro reconstitution of the Escherichia coli 70S ribosome with a full set of recombinant ribosomal proteins
Source: J Biochem. 2021 Nov 8;171(2):227–37. doi: 10.1093/jb/mvab121 (PMC8863084; doi:10.1093/jb/mvab121)
Supplement: Web_Material_mvab121 [file web_material_mvab121.zip › jb-21-10-0317-File008_supplementar figs.docx]

**Supplementary figures and legends**


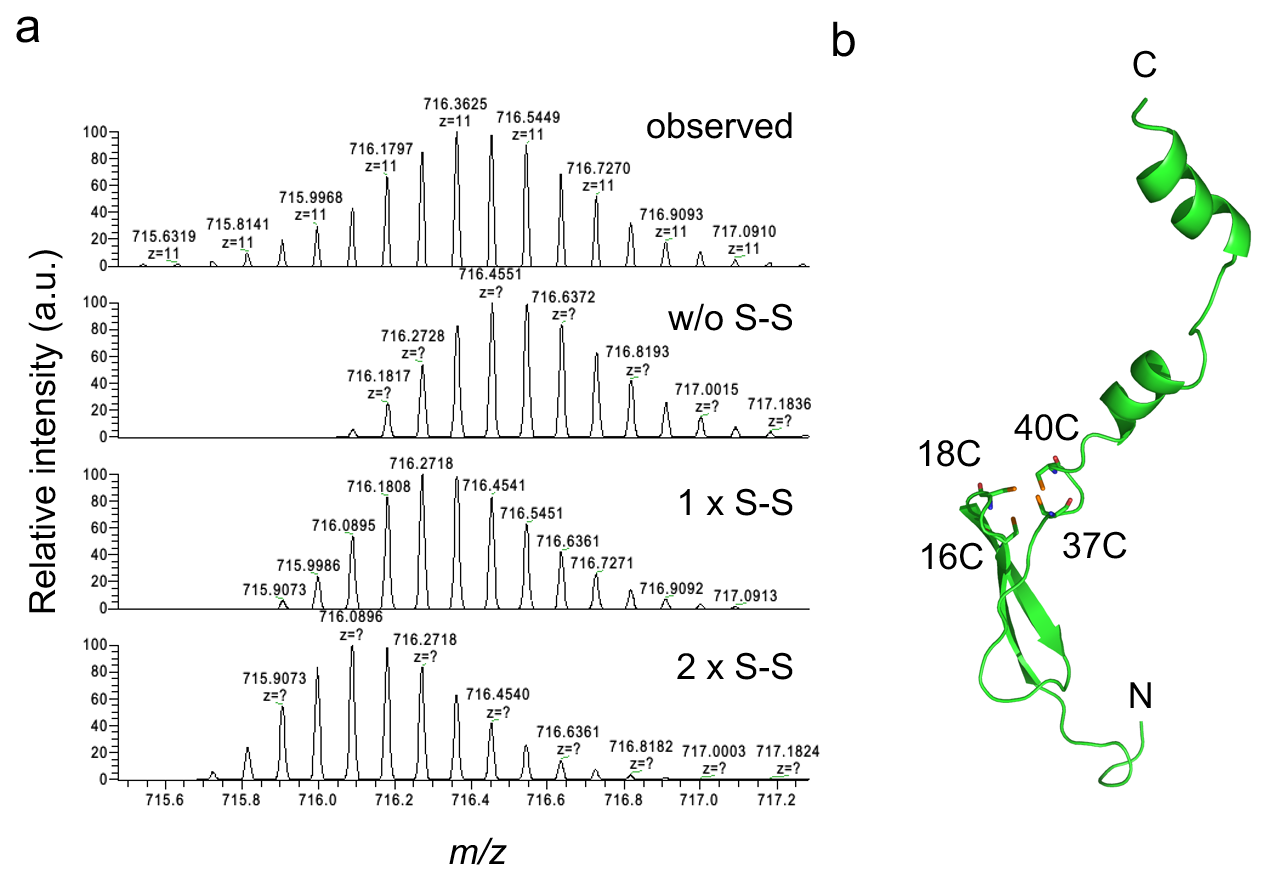


**Supplementary Figure 1. Native MS analysis of bL31.** (a) Native MS analysis was performed for bL31, in which observed mass spectra (observed) and calculated mass spectra without disulfide bonds (w/o S-S), with a single disulfide bond (1 x S-S), and with double single disulfide bonds (2 x S-S) are shown. (b) Three-dimensional structure of bL31 binding to the ribosome, derived from PDB entry 6BU8 (*57*).


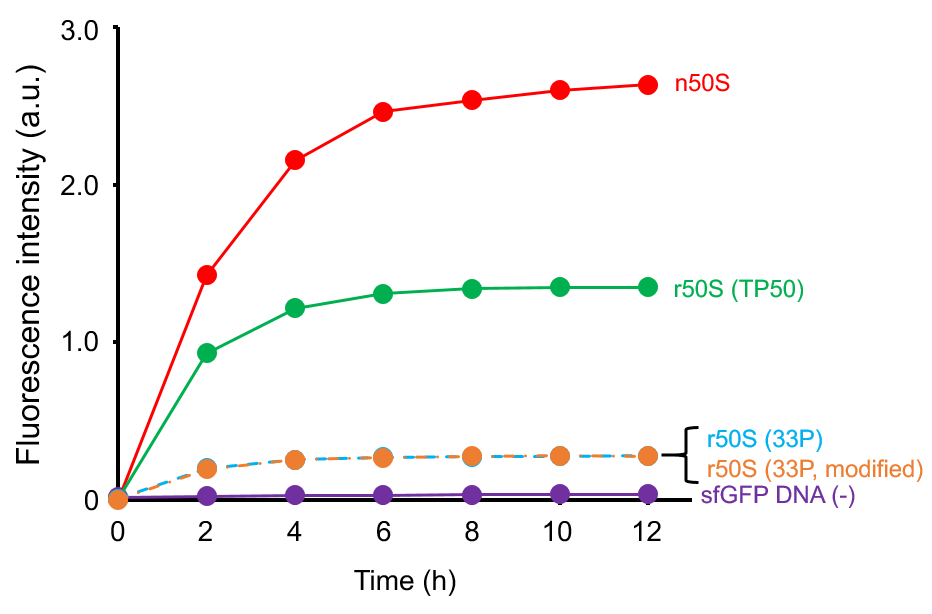


**Supplementary Figure 2. Protein synthesis activities of reconstituted 50S subunits using post-translationally modified uL3 and uL11.** Protein synthesis activities of native 50S subunits (n50S), reconstituted subunits with TP50 (r50S (TP50)), 33 recombinant ribosomal proteins (r50S (33P)), and 33 proteins including post-translationally modified uL3 and uL11 (r50S (33P, modified)) were measured by performing sfGFP synthesis in the PURE system. Time-lapse changes of fluorescence intensity of synthesized sfGFP are shown.


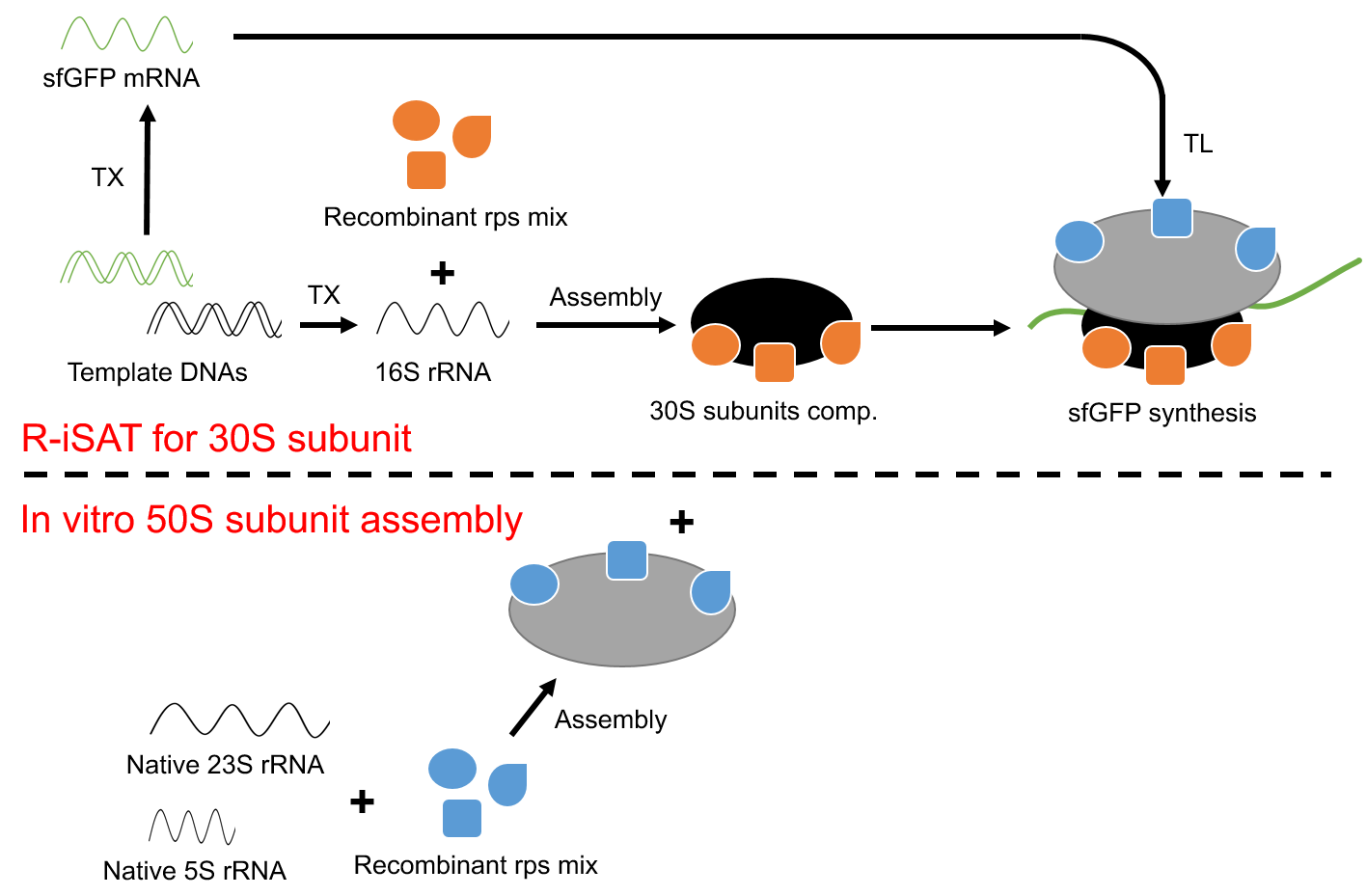


**Supplementary Figure 3. Schematic of integration of in vitro 50S subunit assembly with previously developed R-iSAT.** Two template DNAs encoding 16S rRNA and sfGFP were added into the PURE system. Recombinant ribosomal proteins for 30S subunit bind to the transcribed 16S rRNA and they are assembled into 30S subunits. In vitro 50S subunit assembly is performed in another reaction mixture and purified assembled 50S subunits were added into the R-iSAT reaction mixture, where assembled 30S subunits bind with the assembled 50S subunits for sfGFP synthesis.

**Supplementary Table**

**Supplementary Table S1. List of ribosomal proteins for 50S subunit.** Gene names, original and applied gene and protein sequences, and monoisotopic molecular weight and observed naïve mass values are listed in the table.

**Supplementary Data**

**Supplementary Data 1. DNA sequences of the plasmids used in this study.**

**Supplementary Data 2. Detailed protocol of the purification of ribosomal proteins.** Purification protocols and buffer compositions are listed.

**Supplementary Data 3. Native MS analysis of recombinant ribosomal proteins.** Native MS analysis was performed for uL1-bL36 and observed representative mass spectra with specific charges (upper panel) and calculated mass spectra (lower panel) are shown.

**Supplementary Data 4. Deconvoluted results of native MS analysis of recombinant ribosomal proteins.** Native MS analysis data were analyzed with an available software (*36*) for deconvolution and the data sets including the information of target proteins, categorized in same ID with various charge distributions, are shown. The data corresponding to the mass spectra shown in **Supplementary Data 3** are highlighted in yellow.

**Supplementary Data 5. Post-translational modification analysis with Native MS.** Native MS analysis was performed for uL3, uL11, bL12, uL16, and bL33, which are known to be post-translationally modified. Observed mass spectra (Experimental) and calculated mass spectra of possible forms (Theoretical) are shown.

**Supplementary Data 6. Proteome analysis of ribosomal proteins.** Summary of LC-MS analysis of the ribosomal proteins, which was derived with Proteome Discoverer 2.2 software (Thermo Scientific) is shown.

**Supplementary Data 7. LC-MS analysis of reconstituted 50S subunits.** Signal intensities of peptide fragments from ribosomal proteins and their normalized ratios are listed. Values of peptides from bL31 are highlighted in yellow.
