# Supplementary material for: In vitro reconstitution of the Escherichia coli 70S ribosome with a full set of recombinant ribosomal proteins
Source: J Biochem. 2021 Nov 8;171(2):227–37. doi: 10.1093/jb/mvab121 (PMC8863084; doi:10.1093/jb/mvab121)
Supplement: Web_Material_mvab121 [file web_material_mvab121.zip › jb-21-10-0317-File010__supplementary Data 1.docx]

**Supplementary Data 1. DNA sequences of the plasmids used in this study.**

Underlined: T7 promoter or T7 terminator sequence.

Blue: leader sequence containing His-tag.

Green: SUMO protein.

Red: ribosomal protein, or LgBiT protein or HiBit peptide to be synthesized.

(xxxxxx represents arbitrary ribosomal protein listed in **Supplementary Table 1**).

Purple: PrmA or PrmB

>ribosomal protein uL1-bL36 fused with His-tagged SUMO protein in pET15b

TAATACGACTCACTATAGGGGAATTGTGAGCGGATAACAATTCCCCTCTAGAAATAATTTTGTTTAACTTTAAGAAGGAGATATACCATGGGCAGCAGCCATCATCATCATCATCACAGCAGCGGCATGTCGGACTCAGAAGTCAATCAAGAAGCTAAGCCAGAGGTCAAGCCAGAAGTCAAGCCTGAGACTCACATCAATTTAAAGGTGTCCGATGGATCTTCAGAGATCTTCTTCAAGATCAAAAAGACCACTCCTTTAAGAAGGCTGATGGAAGCGTTCGCTAAAAGACAGGGTAAGGAAATGGACTCCTTAAGATTCTTGTACGACGGTATTAGAATTCAAGCTGATCAGACCCCTGAAGATTTGGACATGGAGGATAACGATATTATTGAGGCTCACAGAGAACAGATTGGTGGTxxxxxxCATATGCCCGGGCTCGAGGGACCCCGCGGGCGGCCGCGTCGACGGATCCGGCTGCTAACAAAGCCCGAAAGGAAGCTGAGTTGGCTGCTGCCACCGCTGAGCAATAACTAGCATAACCCCTTGGGGCCTCTAAACGGGTCTTGAGGGGTTTTTTG

>ribosomal protein uL3 fused with His-tagged SUMO protein co-expressed with PrmB in pET-15b

TAATACGACTCACTATAGGGGAATTGTGAGCGGATAACAATTCCCCTCTAGAAATAATTTTGTTTAACTTTAAGAAGGAGATATACCATGGGCAGCAGCCATCATCATCATCATCACAGCAGCGGCATGTCGGACTCAGAAGTCAATCAAGAAGCTAAGCCAGAGGTCAAGCCAGAAGTCAAGCCTGAGACTCACATCAATTTAAAGGTGTCCGATGGATCTTCAGAGATCTTCTTCAAGATCAAAAAGACCACTCCTTTAAGAAGGCTGATGGAAGCGTTCGCTAAAAGACAGGGTAAGGAAATGGACTCCTTAAGATTCTTGTACGACGGTATTAGAATTCAAGCTGATCAGACCCCTGAAGATTTGGACATGGAGGATAACGATATTATTGAGGCTCACAGAGAACAGATTGGTGGTATGATTGGTTTAGTCGGTAAAAAAGTGGGTATGACCCGTATCTTCACAGAAGACGGCGTTTCTATCCCAGTAACCGTAATCGAAGTTGAAGCAAACCGCGTTACTCAGGTTAAAGACCTGGCTAACGATGGCTACCGTGCTATTCAGGTGACCACCGGTGCTAAAAAAGCTAACCGTGTGACCAAGCCTGAAGCTGGCCACTTCGCTAAAGCTGGCGTAGAAGCTGGCCGTGGTCTGTGGGAATTCCGCCTGGCTGAAGGCGAAGAGTTCACTGTAGGTCAGAGCATTAGCGTTGAACTGTTTGCTGACGTTAAAAAAGTTGACGTAACTGGCACCTCTAAAGGTAAAGGTTTCGCAGGTACCGTTAAGCGCTGGAACTTCCGTACCCAGGACGCTACTCACGGTAACTCCTTGTCTCACCGCGTTCCGGGTTCTATCGGTCAGAACCAGACTCCGGGCAAAGTGTTCAAAGGCAAGAAAATGGCAGGTCAGATGGGTAACGAACGTGTAACCGTTCAGAGCCTTGACGTAGTACGCGTTGACGCTGAGCGCAACCTGCTGCTGGTTAAAGGTGCTGTCCCGGGTGCAACCGGTAGCGACCTGATCGTTAAACCAGCTGTGAAGGCGTAACATATGCCCGGGCTCAATAATTTTGTTTAACTTTAAGAAGGAGATATACATGTGGATAAAATTTTCGTTGATGAAGCAGTAAATGAGCTGCAAACCATTCAGGACATGTTGCGCTGGTCGGTGAGCCGCTTCAGCGCGGCAAATATCTGGTACGGTCACGGTACCGATAACCCGTGGGATGAAGCCGTACAGCTGGTGTTGCCTTCGCTCTACCTGCCGCTGGATATTCCGGAAGATATGCGCACCGCGCGTCTGACCTCCAGCGAAAAACACCGTATTGTTGAACGCGTGATCCGCCGCGTCAATGAACGCATTCCGGTGGCTTACCTGACCAACAAAGCGTGGTTCTGCGGCCATGAATTTTACGTCGATGAACGCGTGCTGGTGCCGCGCTCGCCGATTGGTGAACTGATCAACAATAAATTTGCCGGACTTATCAGCAAGCAACCGCAGCATATTTTAGATATGTGTACTGGTAGCGGCTGCATCGCCATTGCCTGTGCTTATGCCTTCCCGGATGCAGAAGTCGACGCGGTGGATATCTCTCCAGACGCGCTGGCGGTTGCTGAACAGAACATCGAAGAACACGGTCTGATCCACAACGTCATTCCGATTCGTTCCGATCTGTTCCGCGACTTGCCGAAAGTGCAGTACGACCTGATTGTCACTAACCCGCCGTATGTCGATGCGGAAGATATGTCCGACCTGCCAAACGAATACCGCCACGAGCCGGAACTGGGCCTGGCATCTGGCACTGACGGCCTGAAACTGACGCGTCGCATTCTCGGTAACGCGGCAGATTACCTTGCTGATGATGGCGTGTTGATTTGTGAAGTCGGCAACAGCATGGTACATCTTATGGAACAATATCCGGATGTTCCGTTCACCTGGCTGGAGTTTGATAACGGCGGCGATGGTGTGTTTATGCTCACCAAAGAGCAGCTTATTGCCGCACGAGAACATTTCGCGATTTATAAAGATTAACGGATCCGGCTGCTAACAAAGCCCGAAAGGAAGCTGAGTTGGCTGCTGCCACCGCTGAGCAATAACTAGCATAACCCCTTGGGGCCTCTAAACGGGTCTTGAGGGGTTTTTTG

>ribosomal protein uL11 fused with His-tagged SUMO protein co-expressed with PrmA in pET-15b

TAATACGACTCACTATAGGGGAATTGTGAGCGGATAACAATTCCCCTCTAGAAATAATTTTGTTTAACTTTAAGAAGGAGATATACCATGGGCAGCAGCCATCATCATCATCATCACAGCAGCGGCATGTCGGACTCAGAAGTCAATCAAGAAGCTAAGCCAGAGGTCAAGCCAGAAGTCAAGCCTGAGACTCACATCAATTTAAAGGTGTCCGATGGATCTTCAGAGATCTTCTTCAAGATCAAAAAGACCACTCCTTTAAGAAGGCTGATGGAAGCGTTCGCTAAAAGACAGGGTAAGGAAATGGACTCCTTAAGATTCTTGTACGACGGTATTAGAATTCAAGCTGATCAGACCCCTGAAGATTTGGACATGGAGGATAACGATATTATTGAGGCTCACAGAGAACAGATTGGTGGTGCTAAGAAAGTACAAGCCTATGTCAAGCTGCAGGTTGCAGCTGGTATGGCTAACCCGAGTCCGCCAGTAGGTCCGGCTCTGGGTCAGCAGGGCGTAAACATCATGGAATTCTGCAAAGCGTTCAACGCAAAAACTGATTCCATCGAAAAAGGTCTGCCGATTCCGGTAGTAATCACCGTTTACGCTGACCGTTCTTTCACTTTCGTTACCAAGACCCCGCCGGCAGCAGTTCTGCTGAAAAAAGCGGCTGGTATCAAGTCTGGTTCCGGTAAGCCGAACAAAGACAAAGTGGGTAAAATTTCCCGCGCTCAGCTGCAGGAAATCGCGCAGACCAAAGCTGCCGACATGACTGGTGCCGACATTGAAGCGATGACTCGCTCCATCGAAGGTACTGCACGTTCCATGGGCCTGGTAGTGGAGGACTAACATATGCCCGGGCTCAATAATTTTGTTTAACTTTAAGAAGGAGATATACATATGCCTTGGATCCAACTGAAACTGAACACCACCGGCGCGAACGCGGAAGATCTTAGCGATGCGCTGATGGAAGCGGGTGCCGTTTCTATCACTTTTCAGGATACCCACGATACGCCAGTATTTGAACCGCTGCCGGGCGAAACGCGCCTGTGGGGCGACACCGATGTGATTGGTCTGTTCGACGCTGAAACCGATATGAACGACGTGGTGGCGATTCTGGAAAACCATCCGCTGCTCGGCGCAGGCTTCGCGCATAAAATCGAACAACTAGAAGATAAAGACTGGGAGCGCGAATGGATGGATAATTTCCACCCGATGCGCTTTGGTGAACGACTGTGGATCTGCCCTAGCTGGCGTGATGTGCCGGACGAAAACGCCGTCAACGTGATGTTAGATCCAGGGCTGGCGTTTGGTACGGGTACCCATCCAACCACCTCTCTGTGCCTGCAATGGCTCGACAGCCTCGATTTAACCGGTAAAACAGTCATCGACTTTGGCTGTGGTTCCGGCATTCTGGCGATCGCGGCGCTGAAACTGGGTGCAGCAAAAGCCATTGGTATTGATATCGATCCGCAGGCGATTCAGGCCAGCCGCGATAACGCCGAACGTAATGGCGTTTCTGACCGTCTGGAACTCTACTTACCGAAAGATCAGCCAGAAGAAATGAAAGCCGACGTGGTGGTCGCTAACATCCTTGCAGGCCCATTACGTGAACTGGCACCGTTAATCAGCGTCCTGCCGGTTTCAGGCGGTTTGCTGGGCCTTTCCGGTATTCTGGCAAGCCAGGCAGAGAGCGTTTGTGAAGCTTATGCCGATAGCTTCGCACTGGACCCGGTCGTGGAAAAAGAAGAGTGGTGCCGTATTACCGGTCGTAAGAATTAACGGATCCGGCTGCTAACAAAGCCCGAAAGGAAGCTGAGTTGGCTGCTGCCACCGCTGAGCAATAACTAGCATAACCCCTTGGGGCCTCTAAACGGGTCTTGAGGGGTTTTTTG

> LgBiT fused with His-tagged SUMO protein in pET15b

TAATACGACTCACTATAGGGGAATTGTGAGCGGATAACAATTCCCCTCTAGAAATAATTTTGTTTAACTTTAAGAAGGAGATATACCATGGGCAGCAGCCATCATCATCATCATCACAGCAGCGGCATGTCGGACTCAGAAGTCAATCAAGAAGCTAAGCCAGAGGTCAAGCCAGAAGTCAAGCCTGAGACTCACATCAATTTAAAGGTGTCCGATGGATCTTCAGAGATCTTCTTCAAGATCAAAAAGACCACTCCTTTAAGAAGGCTGATGGAAGCGTTCGCTAAAAGACAGGGTAAGGAAATGGACTCCTTAAGATTCTTGTACGACGGTATTAGAATTCAAGCTGATCAGACCCCTGAAGATTTGGACATGGAGGATAACGATATTATTGAGGCTCACAGAGAACAGATTGGTGGTATGGTCTTCACACTCGAAGATTTCGTTGGGGACTGGGAACAGACAGCCGCCTACAACCTGGACCAAGTCCTTGAACAGGGAGGTGTGTCCAGTTTGCTGCAGAATCTCGCCGTGTCCGTAACTCCGATCCAAAGGATTGTCCGGAGCGGTGAAAATGCCCTGAAGATCGACATCCATGTCATCATCCCGTATGAAGGTCTGAGCGCCGACCAAATGGCCCAGATCGAAGAGGTGTTTAAGGTGGTGTACCCTGTGGATGATCATCACTTTAAGGTGATCCTGCCCTATGGCACACTGGTAATCGACGGGGTTACGCCGAACATGCTGAACTATTTCGGACGGCCGTATGAAGGCATCGCCGTGTTCGACGGCAAAAAGATCACTGTAACAGGGACCCTGTGGAACGGCAACAAAATTATCGACGAGCGCCTGATCACCCCCGACGGCTCCATGCTGTTCCGAGTAACCATCAACAGTTAACATATGCCCGGGCTCGAGGGACCCCGCGGGCGGCCGCGTCGACGGATCCGGCTGCTAACAAAGCCCGAAAGGAAGCTGAGTTGGCTGCTGCCACCGCTGAGCAATAACTAGCATAACCCCTTGGGGCCTCTAAACGGGTCTTGAGGGGTTTTTTG

> MIIIIGSSG-HiBiT-tag in pET-15b

TAATACGACTCACTATAGGGGAATTGTGAGCGGATAACAATTCCCCTCTAGAAATAATTTTGTTTAACTTTAAGAAGGAGATATACCATGATTATTATTATTGGTTCTTCTGGTGTTTCTGGTTGGCGGCTGTTCAAGAAGATTTCTTAAGGATCCGGCTGCTAACAAAGCCCGAAAGGAAGCTGAGTTGGCTGCTGCCACCGCTGAGCAATAACTAGCATAACCCCTTGGGGCCTCTAAACGGGTCTTGAGGGGTTTTTTG
