# Supplementary material for: In vitro reconstitution of the Escherichia coli 70S ribosome with a full set of recombinant ribosomal proteins
Source: J Biochem. 2021 Nov 8;171(2):227–37. doi: 10.1093/jb/mvab121 (PMC8863084; doi:10.1093/jb/mvab121)

**Supplementary Data 3. Native MS analysis of recombinant ribosomal proteins.**

**uL1**


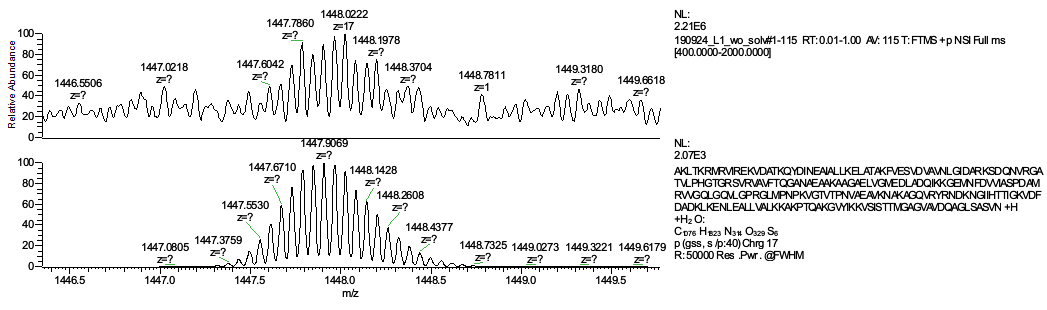


**uL3**


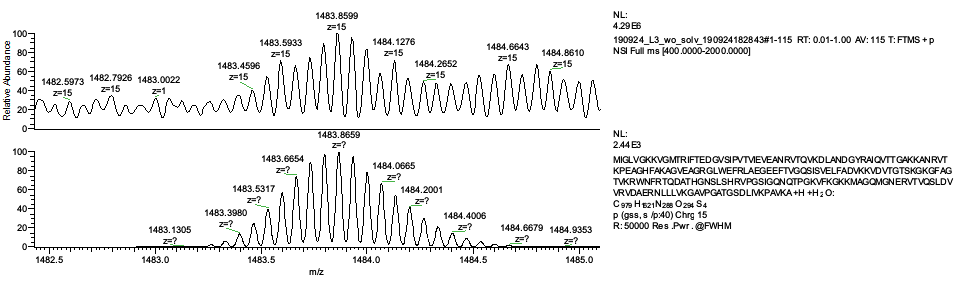


**uL4**


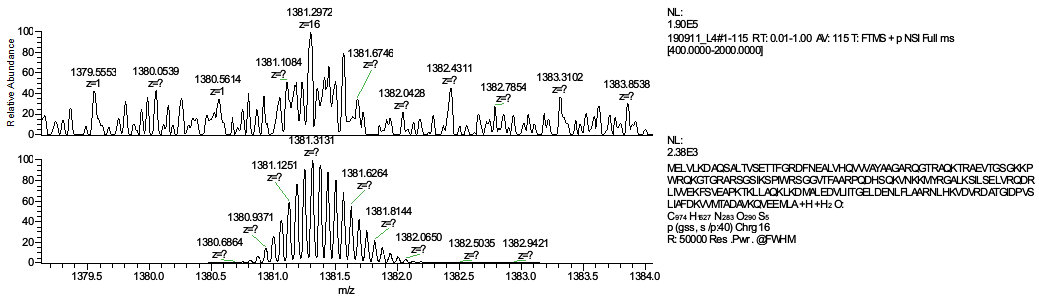


**uL5**


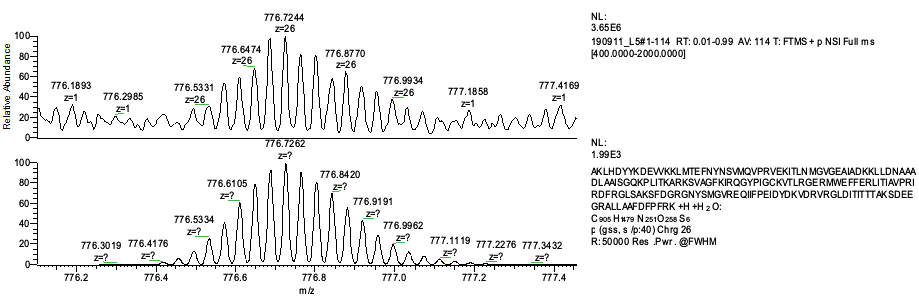


**uL6**


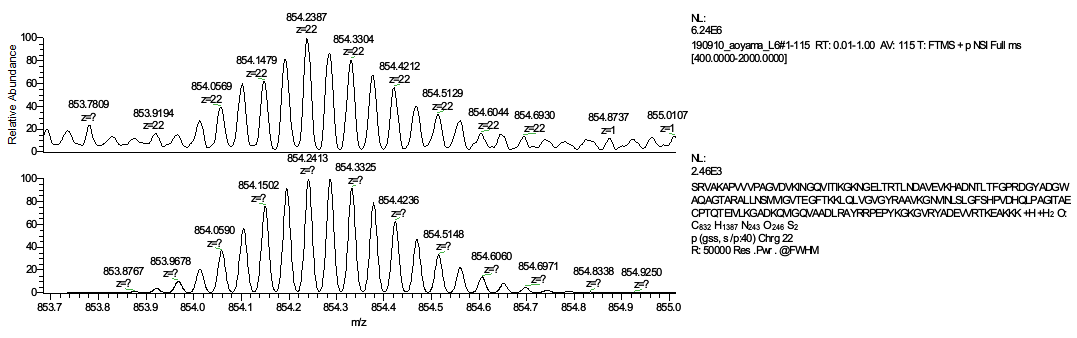


**bL9**


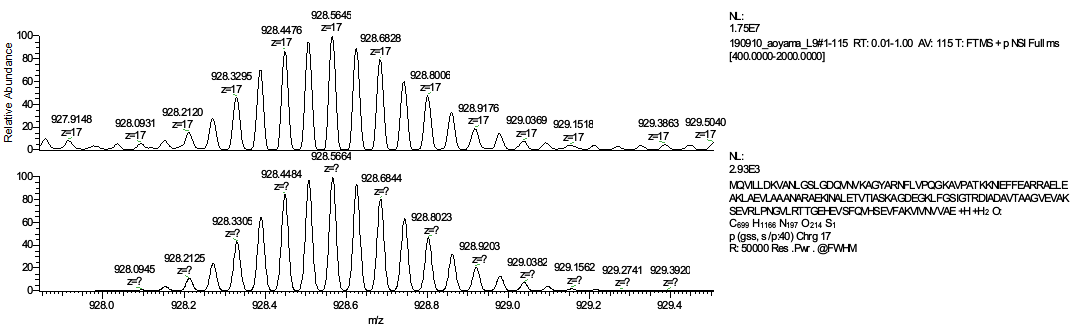


**uL10**


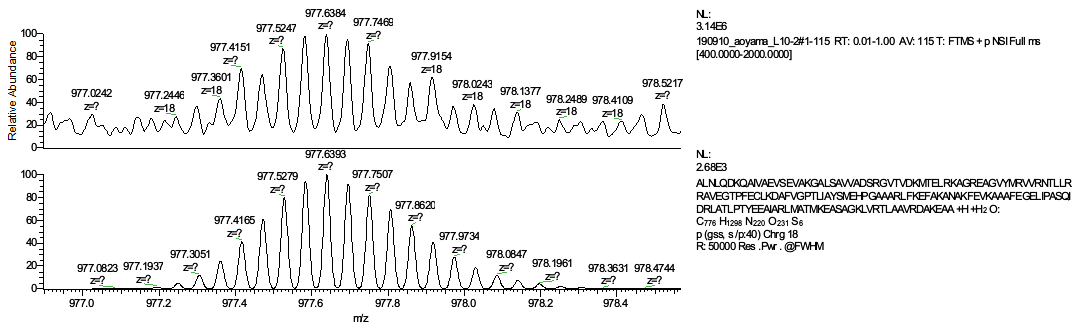


**uL11**


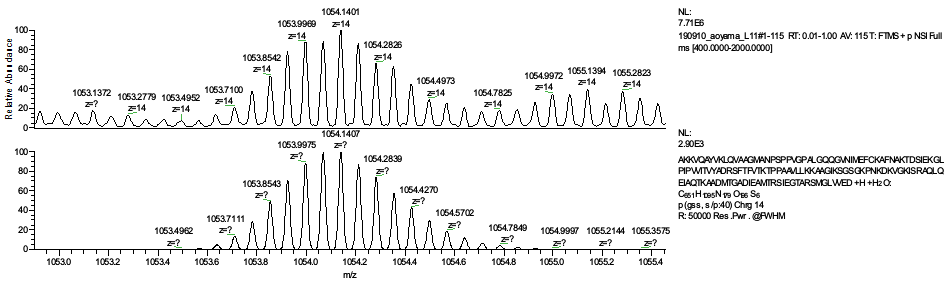


**bL12**


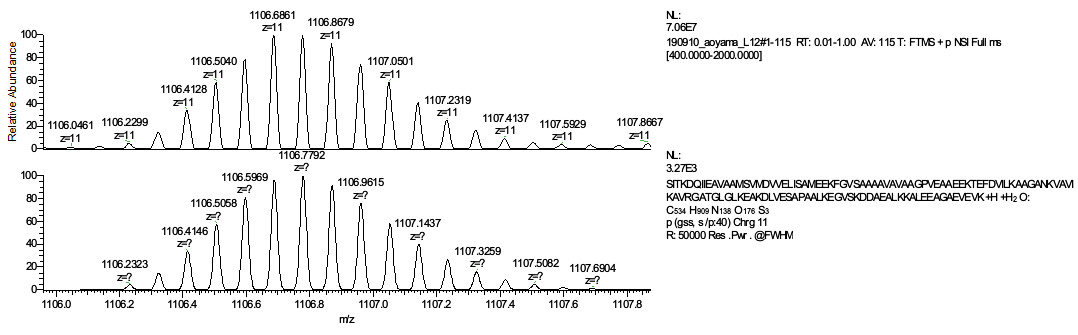


**uL13**


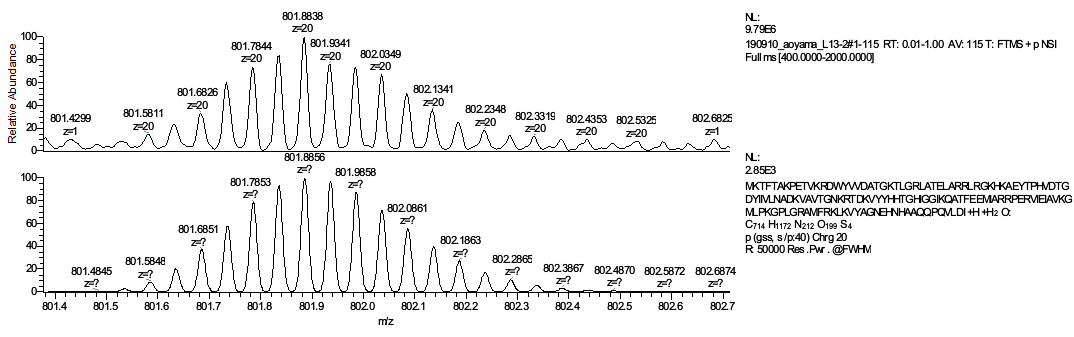


**uL14**


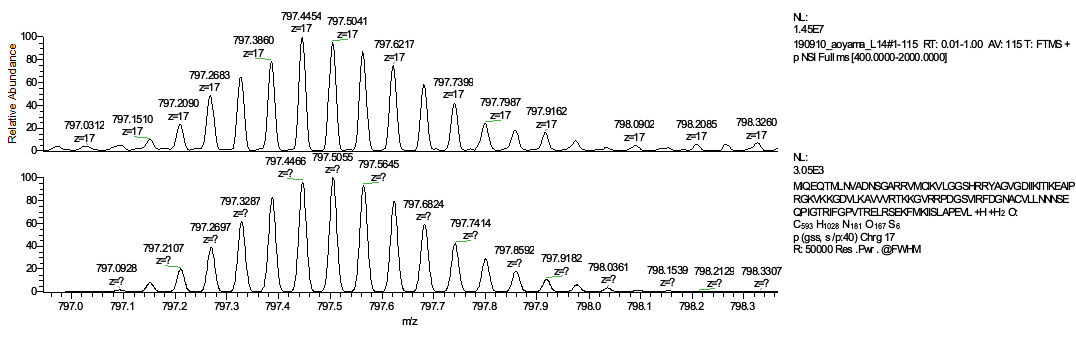


**uL15**


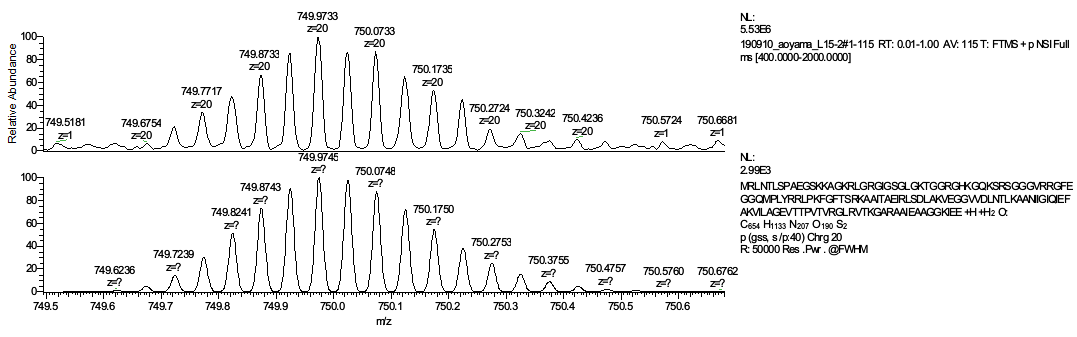


**uL16**


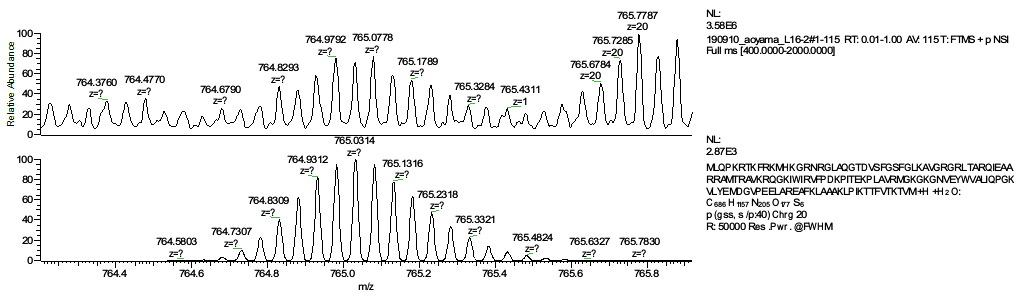


**uL17**


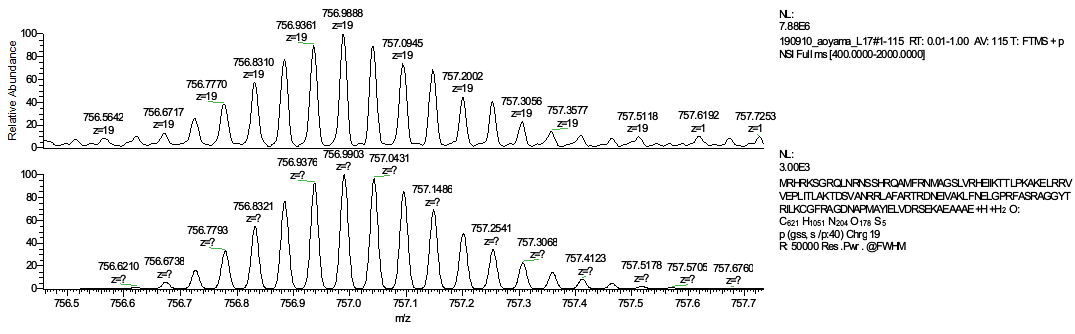


**uL18**


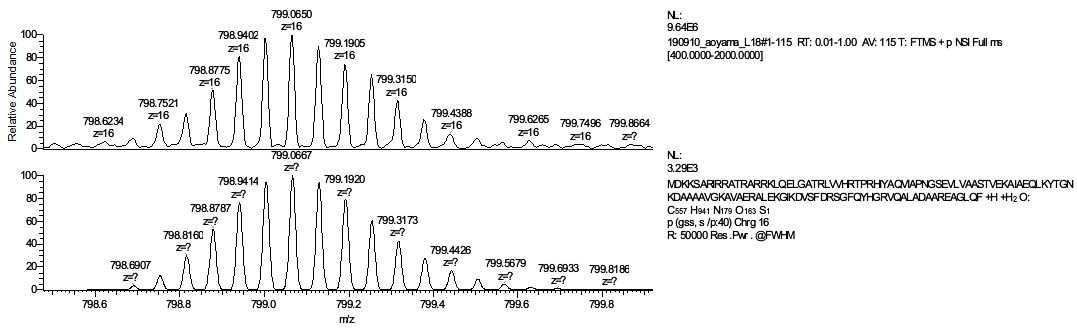


**bL19**


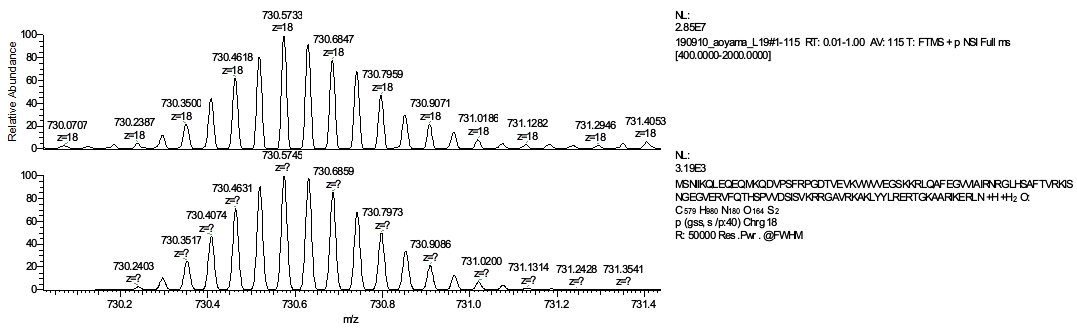


**bL20**


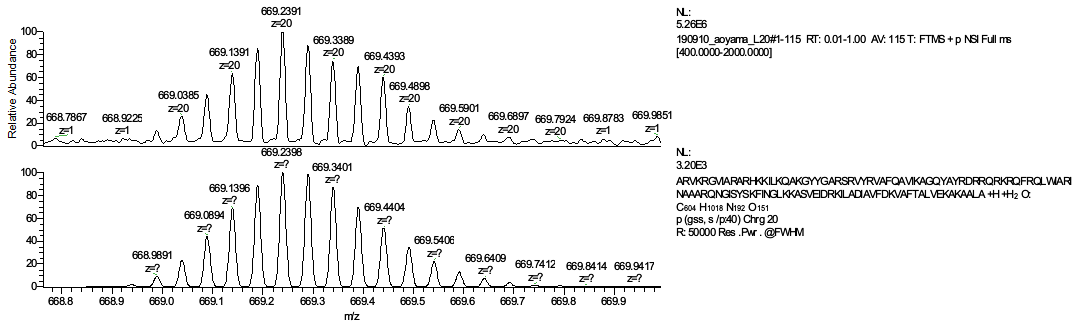


**bL21**


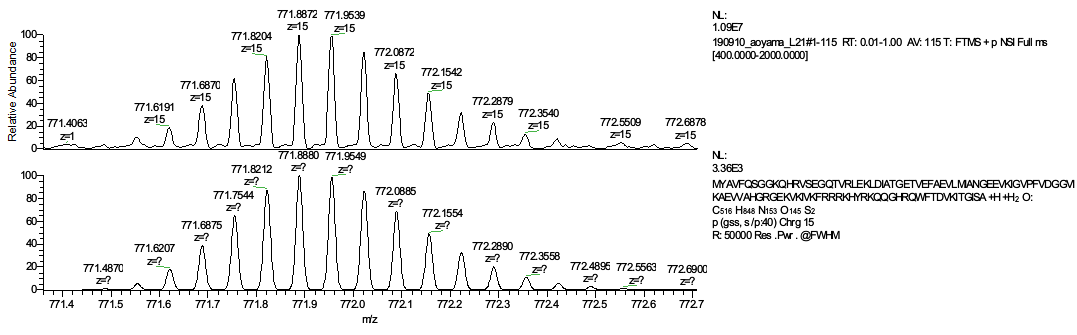


**uL22**


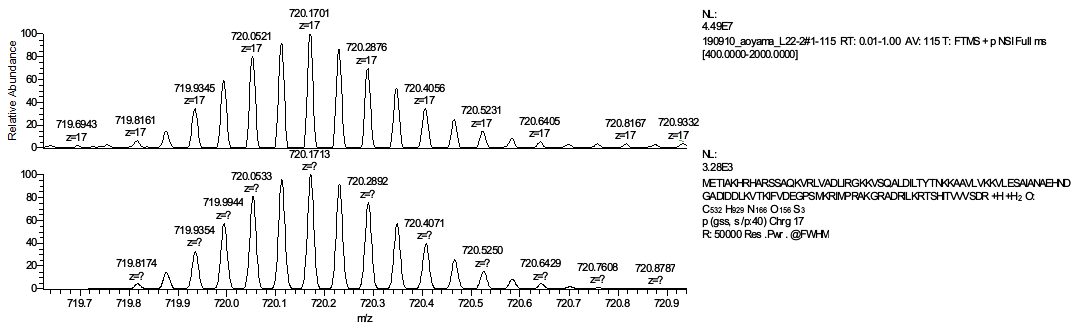


**uL23**


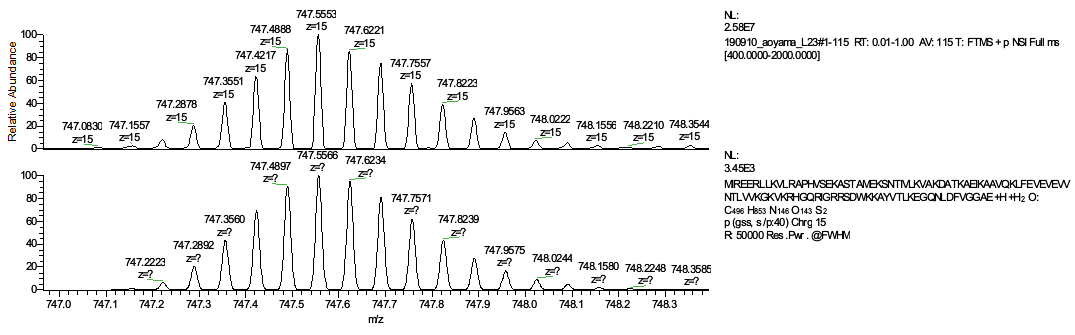


**uL24**


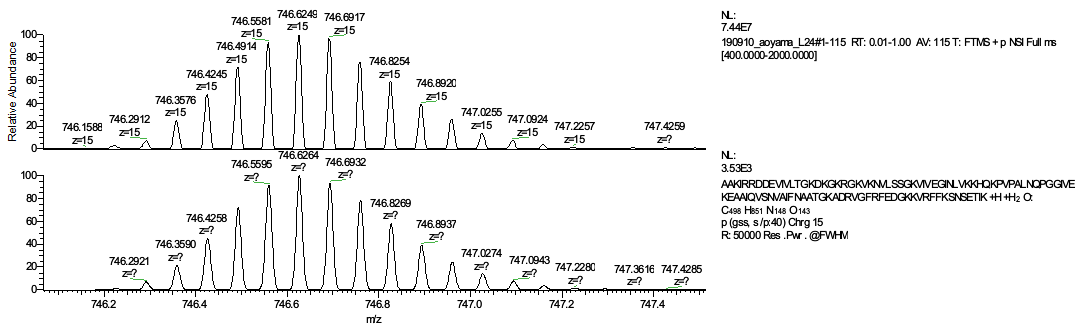


**bL25**


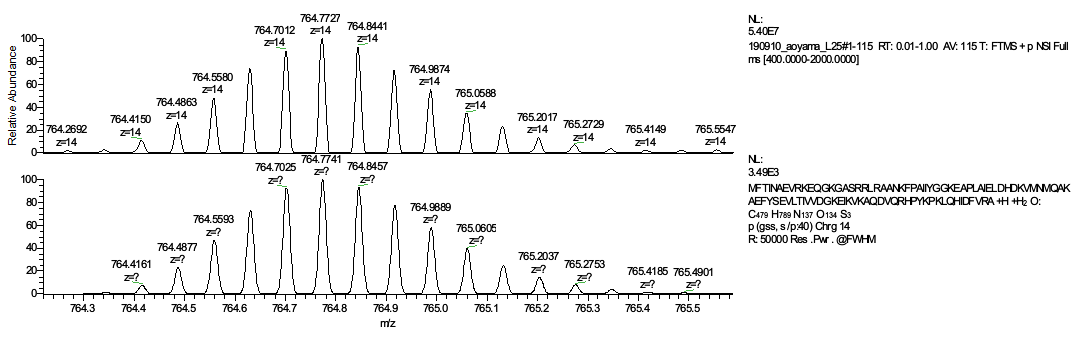


**bL27**


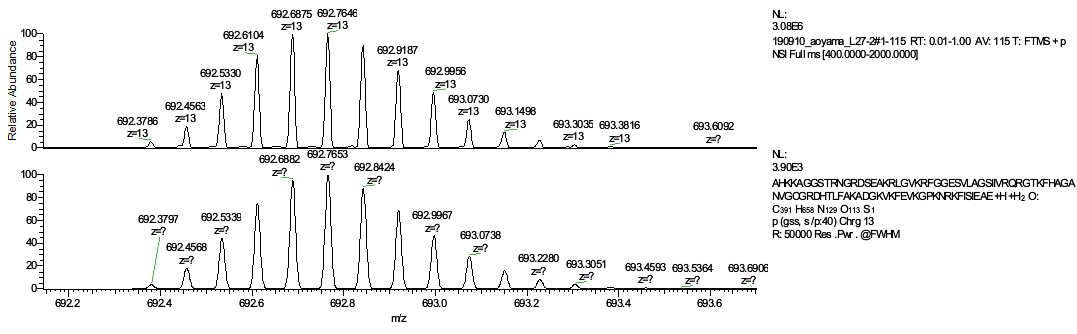


**bL28**


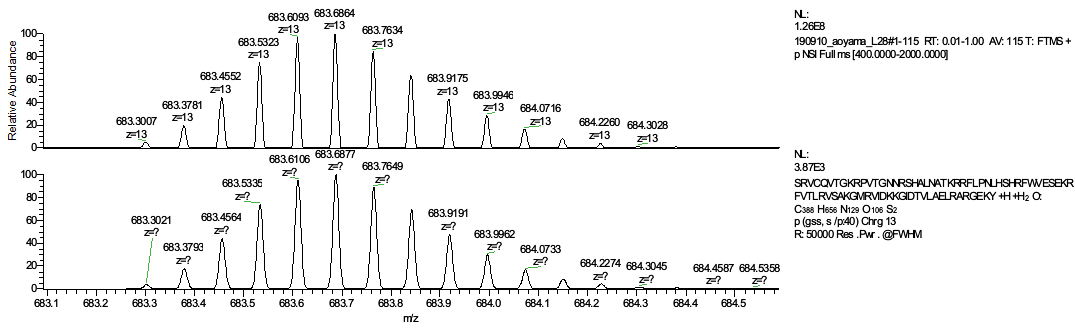


**uL29**


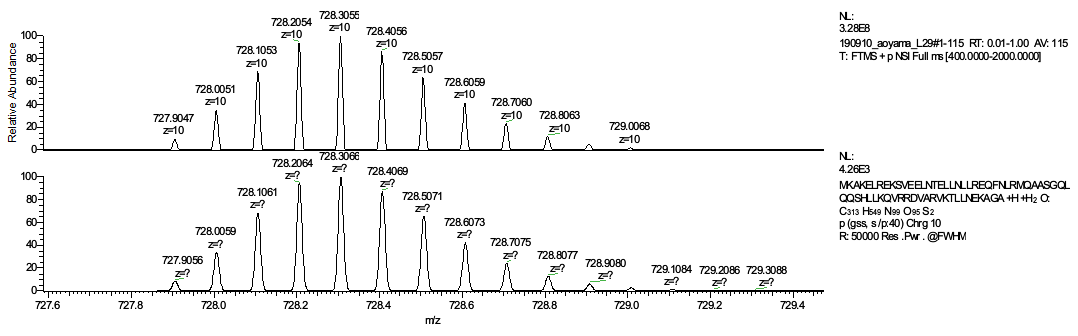


**uL30**


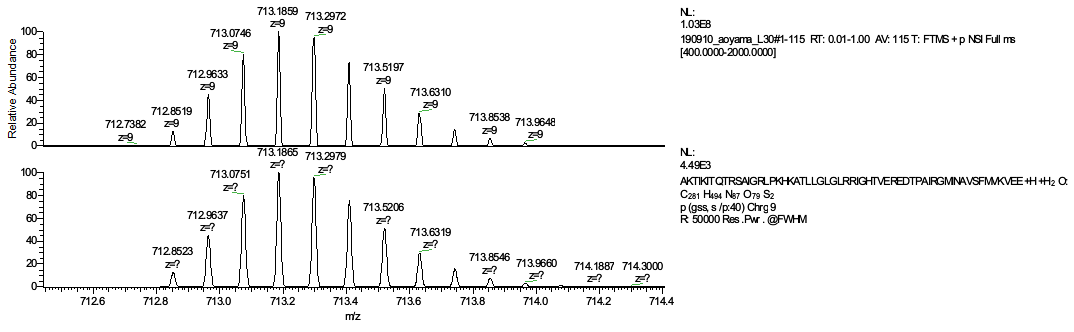


**bL31**


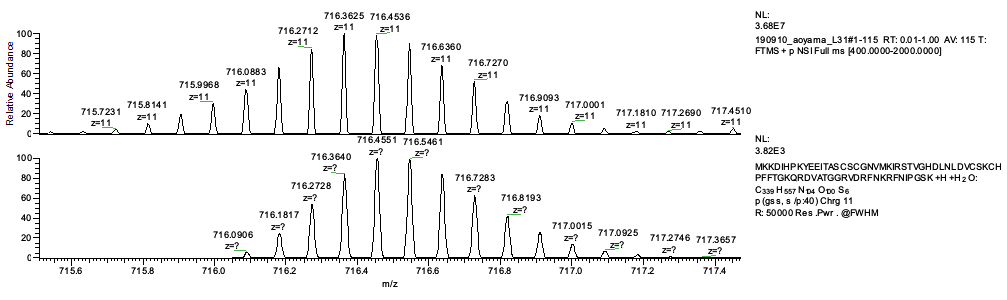


**bL32**


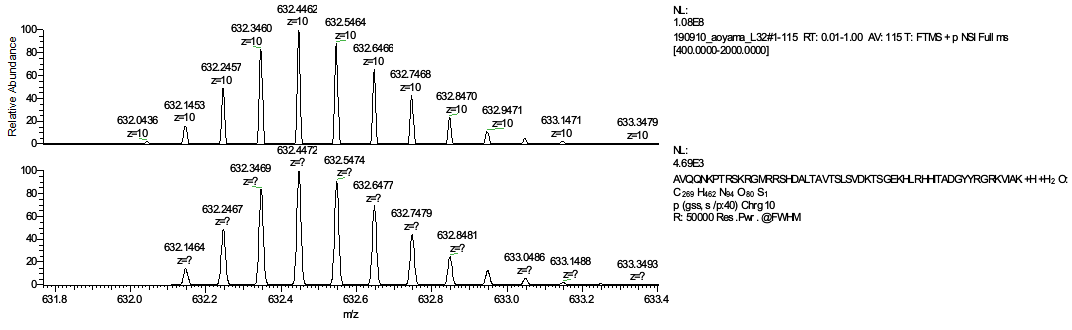


**bL33**


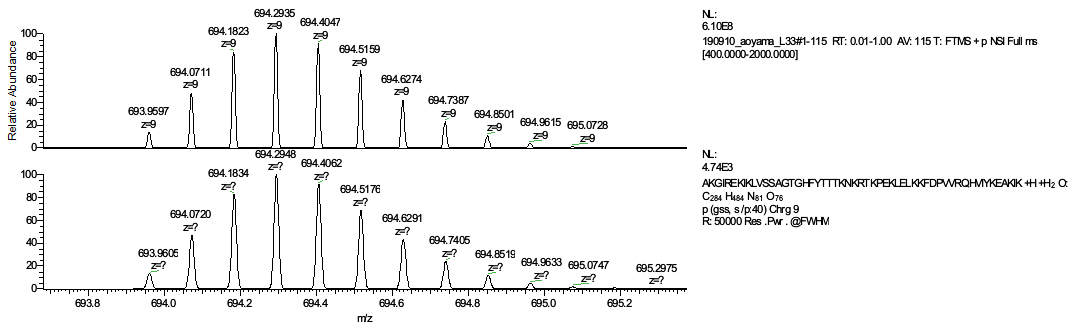


**bL34**


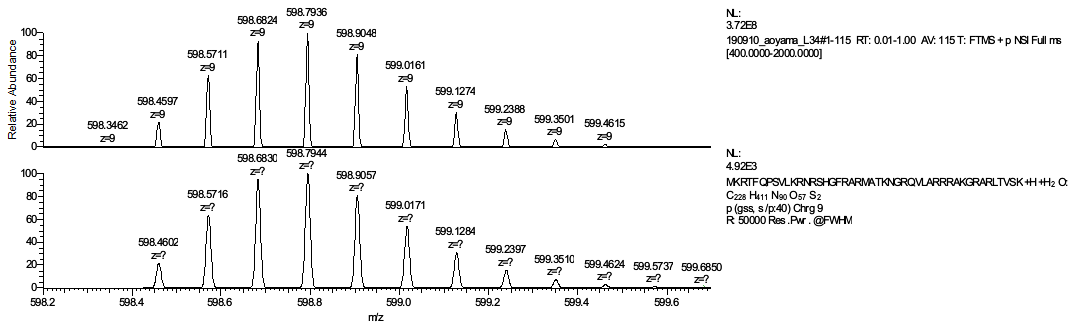


**bL35**


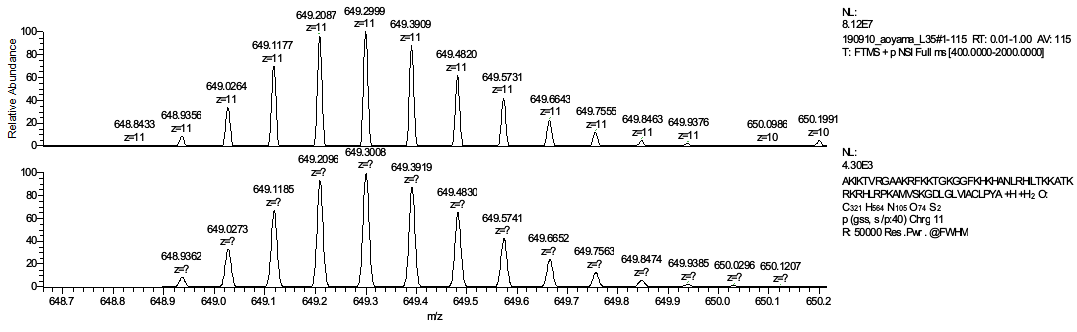


**bL36**


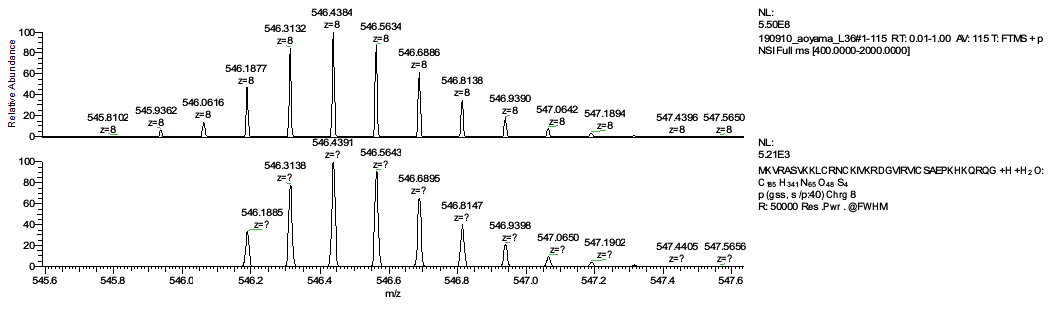

Supplement: Web_Material_mvab121 [file web_material_mvab121.zip › jb-21-10-0317-File012__supplementary Data 3.docx]
