# Supplementary material for: In vitro reconstitution of the Escherichia coli 70S ribosome with a full set of recombinant ribosomal proteins
Source: J Biochem. 2021 Nov 8;171(2):227–37. doi: 10.1093/jb/mvab121 (PMC8863084; doi:10.1093/jb/mvab121)
Supplement: Web_Material_mvab121 [file web_material_mvab121.zip › jb-21-10-0317-File014__supplementary data 5.docx]

**Supplementary Data 5. Post-translational modification analysis with native MS.**

**uL3**


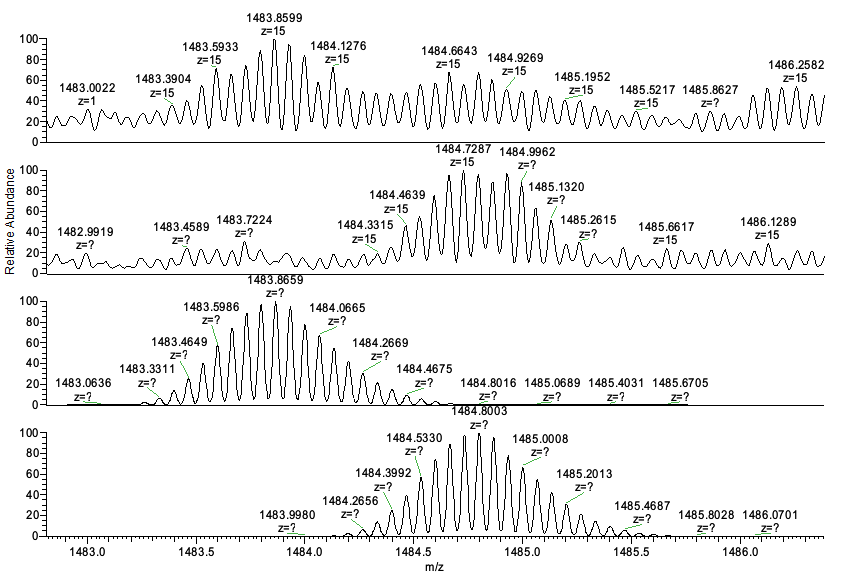


**Theoretical: uL3 + methylation**

**Theoretical: uL3**

**Experimental: uL3 + methylation**

**Experimental: uL3**

**uL11**


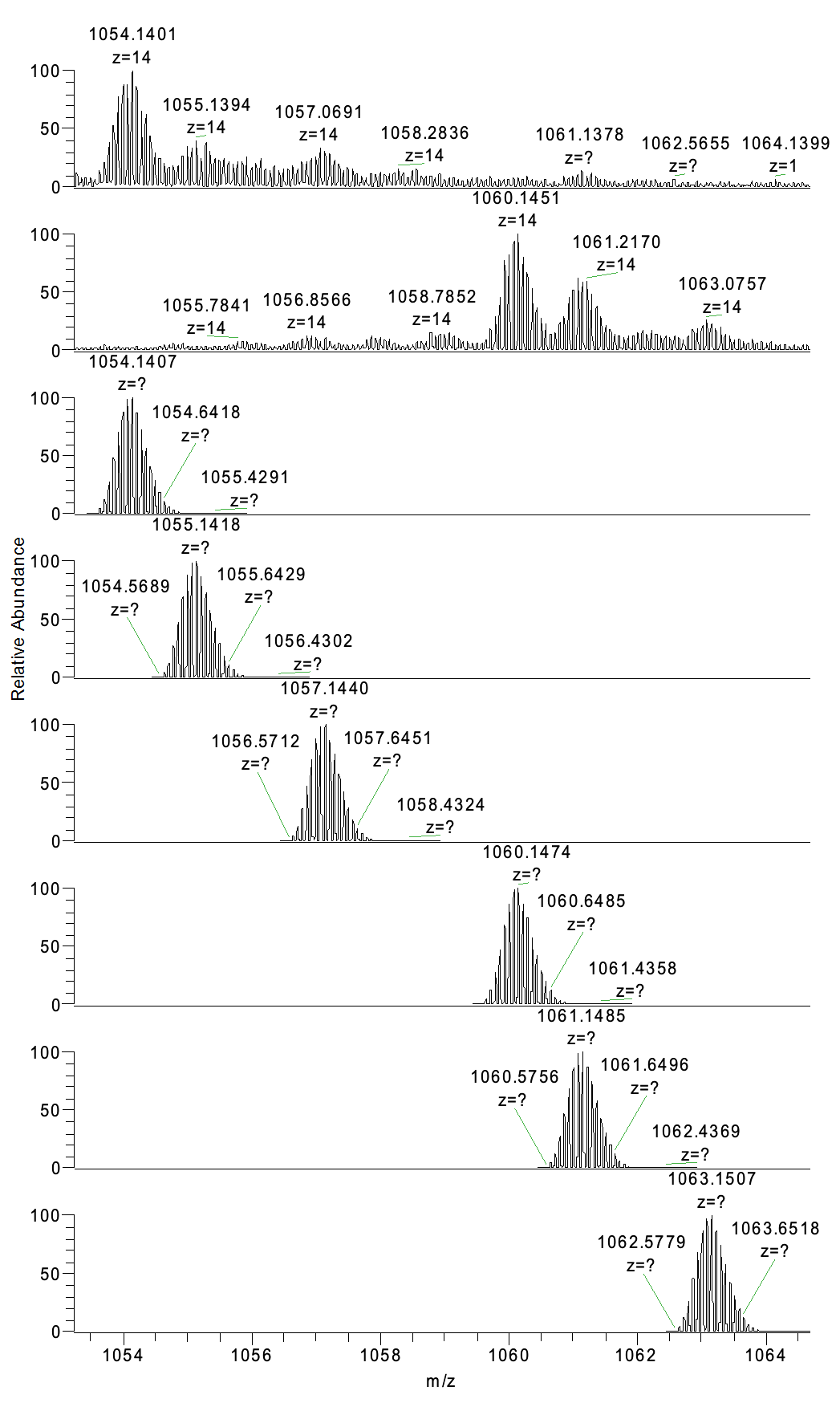


**Theoretical: uL11 + 9 methylation**

**Theoretical: uL11 + 7 methylation**

**Theoretical: uL11 + 6 methylation**

**Theoretical: uL11 + 3 methylation**

**Theoretical: uL11 + 1 methylation**

**Theoretical: uL11**

**Experimental: uL11 + methylation**

**Experimental: uL11**

**bL12**


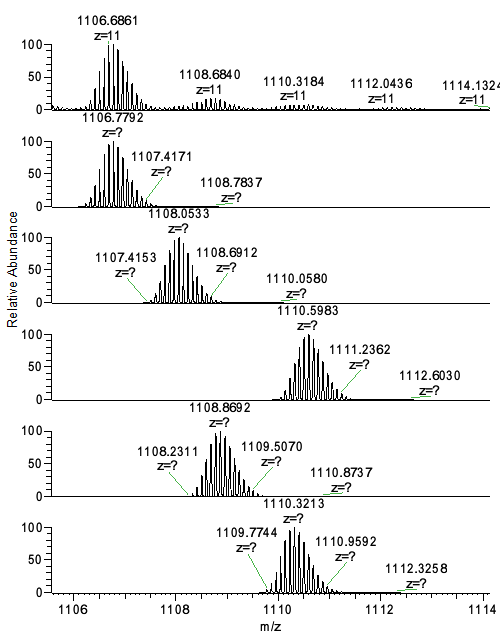


**Theoretical: bL12 + K^+^ adduct**

**Theoretical: bL12 + Na^+^ adduct**

**Theoretical: bL12 + acetylation**

**Theoretical: bL12 + methylation**

**Theoretical: bL12**

**Experimental: bL12**

**uL16**


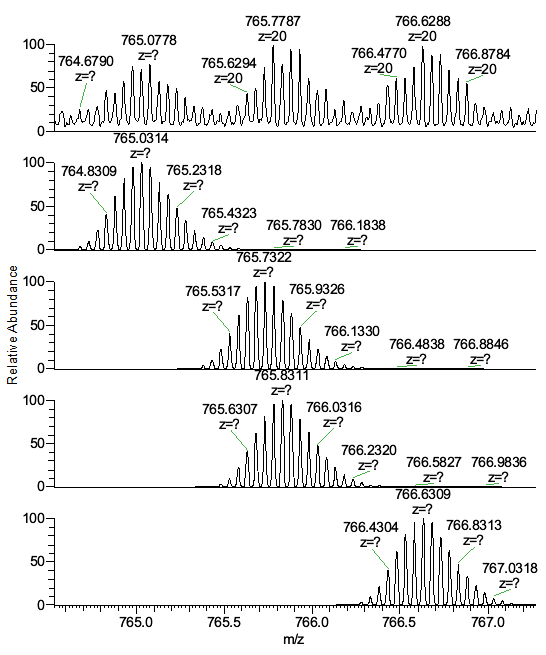


**Theoretical: uL16**

**+ doubly oxidized**

**Theoretical: uL16**

**+ oxidized**

**Theoretical: uL16**

**+ methylation**

**Experimental: uL16**

**Theoretical: uL16**

**bL33**


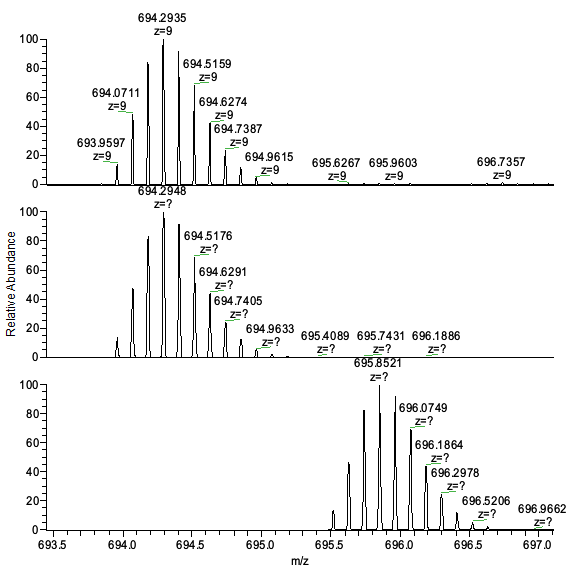


**Theoretical: bL33 + methylation**

**Experimental: bL33**

**Theoretical: bL33**
